# Design and optimization of caspase-1-responsive fluorescent probes for pyroptosis imaging and anti-pyroptosis drug screening

**DOI:** 10.1039/d5sc07690k

**Published:** 2025-11-10

**Authors:** Wei Wang, Guanrui Huang, Yeting Zhou, Yue Wang, Luling Wu, Tony D. James, Weili Wang, Yi Wang

**Affiliations:** a Pharmaceutical Informatics Institute, College of Pharmaceutical Sciences, Zhejiang University Hangzhou 310058 China zjuwangyi@zju.edu.cn; b National Key Laboratory of Chinese Medicine Modernization, Innovation Center of Yangtze River Delta, Zhejiang University Jiaxing 314102 China; c Department of Orthopedic Surgery, The First Affiliated Hospital, Zhejiang University School of Medicine Hangzhou 310003 China; d State Key Laboratory of Analytical Chemistry for Life Science, School of Chemistry and Chemical Engineering, Nanjing University 163 Xianlin Avenue Nanjing 210023 China lulingwu@nju.edu.cn; e Department of Chemistry, University of Bath BA2 7AY UK; f Department of Hepatobiliary and Pancreatic Surgery, The Second Affiliated Hospital, Zhejiang University School of Medicine Hangzhou 310009 China wangweili@zju.edu.cn; g Jinan Microecological Biomedicine Shandong Laboratory Jinan 250118 China; h School of Chemistry and Chemical Engineering, Henan Normal University Xinxiang 453007 China

## Abstract

Pyroptosis is a recently-identified form of inflammatory caspase-dependent programmed cell death that is closely associated with many diseases. Real-time imaging of pyroptosis is crucial for monitoring the inflammatory pathological process. Caspase-1, a representative of inflammatory caspase, plays a pivotal role in pyroptosis and inflammatory diseases. Therefore, caspase-1 activity can reflect pyroptosis and related inflammatory states. Herein, we report on a variety of caspase-1 activatable probes based on potential hydrolytic peptides of caspase-1. Through systematic performance evaluation, we identified that FPy1 designed based on the cleavage of pyroptosis-related protein GSDMD exhibits the best detection performance. Thus, the specific peptide –FLTDG– from GSDMD could serve as a potential responsive element for the design of caspase-1 or pyroptosis-related probes. Owing to the outstanding performance of FPy1, we further applied it to monitor pyroptosis processes in three distinct biological contexts, *i.e.* cellular, cell spheroid, and *in vivo* models, using degenerative bone and joint diseases, *i.e.* intervertebral disc degeneration and osteoarthritis. Moreover, we combined FPy1 with high-content analysis to establish a screening platform for caspase-1 modulators, based on the classic NLRP3 inflammasome-mediated caspase-1 activation model in primary macrophages. Collectively, these results illustrated the potential of FPy1 as a versatile tool for tracking the progression of pyroptosis and monitoring caspase-1 activity across various application scenarios.

## Abstract 2

A novel caspase-1-activatable probe FPy1 has been successfully used for the imaging of caspase-1 or pyroptosis across multiple biological scenarios, encompassing multiscale pyroptosis imaging and high-throughput screening of caspase-1 inhibitors.

## Graphical abstract

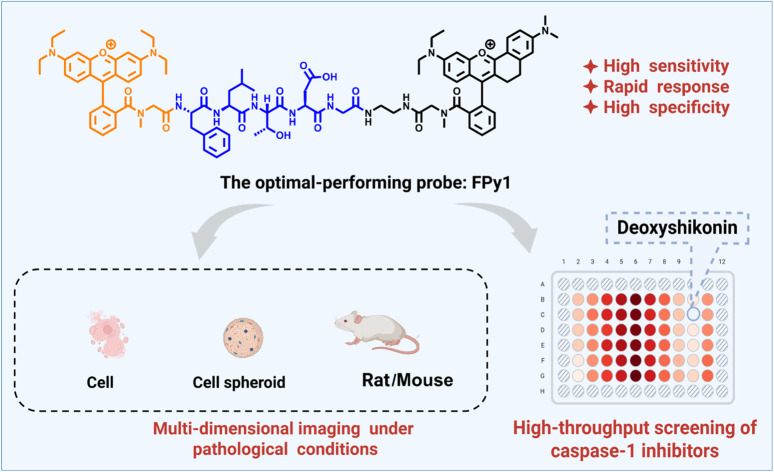

## Introduction

Pyroptosis, a form of programmed cell death distinct from apoptosis and necrosis, was mechanistically defined in 2015 with the discovery of gasdermin D (GSDMD) as its executioner protein.^1^ When pyroptosis occurs, cells undergo several physiological and morphological changes, including plasma membrane rupture, the release of intracellular content and the secretion of inflammatory cytokines, ultimately leading to cell lysis and death.^2,3^ Despite the complex molecular mechanisms underlying pyroptosis, it is widely accepted that inflammatory caspases play a significant role in pyroptosis.^4^ Among these inflammatory caspases, activated caspase-1 (Cas-1) could cleave pore-forming protein gasdermin D (GSDMD) to trigger pyroptosis through the canonical inflammasome pathway. Furthermore, Cas-1 cleaves and processes precursor forms of IL-1β and IL-18, producing activated cytokines.^5^ Mature IL-1β and IL-18 are proinflammatory mediators that activate the immune response and exacerbate inflammation.^6^

Recently, many studies have demonstrated that pyroptosis is closely associated with various diseases, such as viral and bacterial infections,^7^ arthritis,^8^ atherosclerosis,^9^ and intervertebral disc degeneration.^10^ Therefore, monitoring pyroptosis is beneficial for providing additional methods for disease diagnosis and drug screening. To date, multiple techniques have been developed for studying pyroptosis, such as the microscopic observation of cell morphology, and the detection of molecular biomarkers using western blotting or ELISA.^11^ However, these methods are invasive and cumbersome. As such activity-based molecular fluorescent probes represent a valid alternative, since they have been used as non-invasive imaging tools for the real-time monitoring of specific biomarkers in living systems with high selectivity, sensitivity, and good biocompatibility. Therefore, they have been widely applied in disease diagnosis and drug screening.^12–16^ Currently, there are few reports on fluorescent probes for monitoring pyroptosis. Yang *et al.* designed a H_2_O_2_-responsive ratiometric probe for pyroptosis visualization.^17^ While Zhan *et al.* designed a dual-responsive probe for HClO and viscosity to monitor the pyroptosis process.^18^ Mei *et al.* created a dual-responsive probe for H_2_O_2_ and viscosity to characterize pyroptosis.^19^ Zhao *et al.* designed a multifunctional probe that simultaneously tracks fluctuations in mitochondrial polarity and mtDNA to monitor mitochondrial-associated pyroptosis.^20^ However, reactive oxygen species, viscosity and mtDNA can also exhibit abnormal levels in other pathological process, and are not specific biomarkers for pyroptosis. The aforementioned studies were ultimately validated by detecting pyroptosis-executing proteins to confirm the reliability of both cellular models and fluorescent imaging, along with their correlation. Therefore, targeting pyroptosis-specific biomarkers is an essential consideration when designing suitable probes.

**Scheme 1:**
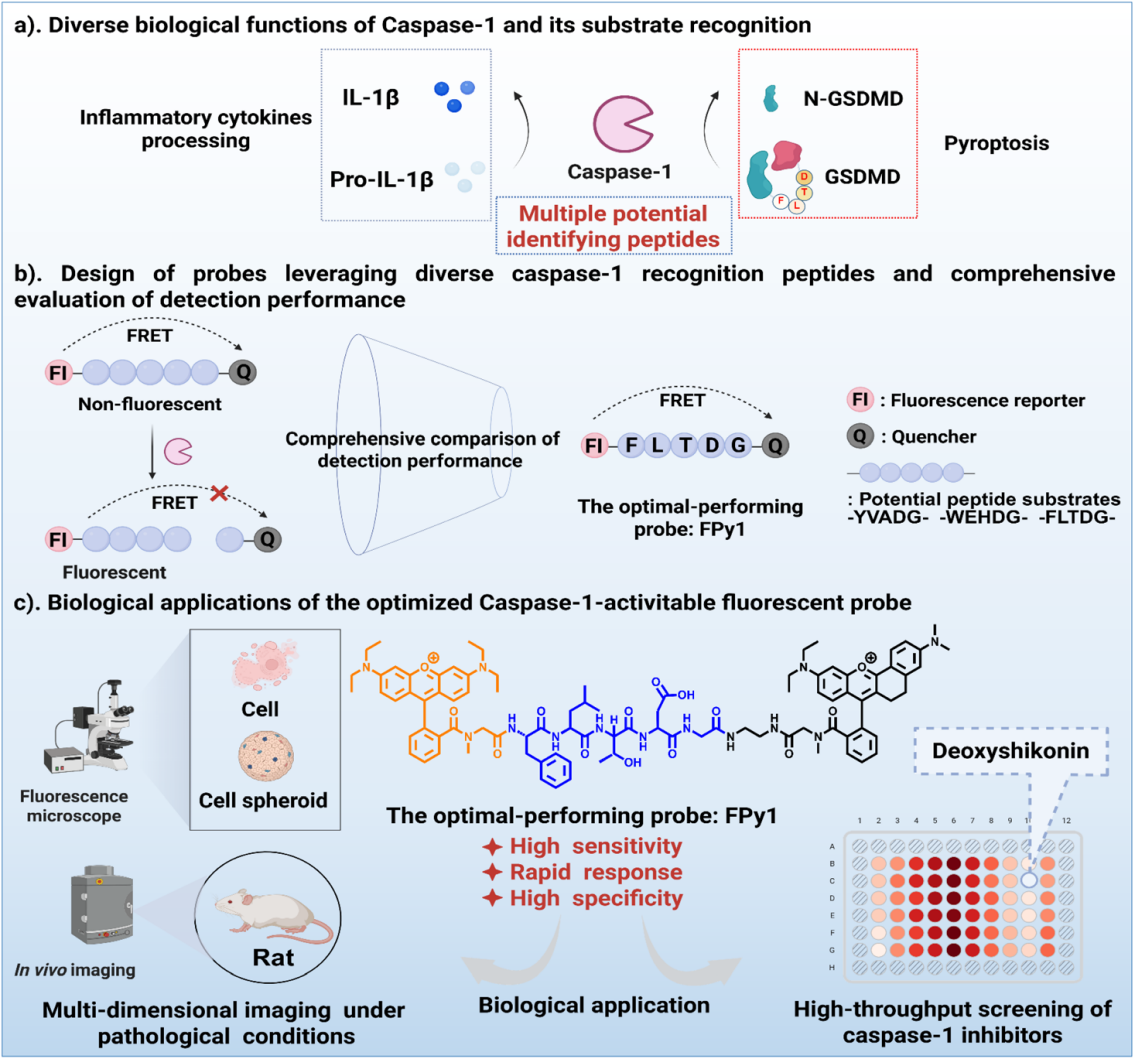
Schematic illustration of rational design and performance screening for multiple caspase-1-activatable probes. (a) The diverse biological functions of caspase-1 under inflammatory pathological conditions and its recognized substrates. (b) Design of probes leveraging diverse caspase-1 recognition peptides and comprehensive evaluation of detection performance. (c) Application of the optimal-performing probe FPy1 in multi-dimensional imaging and assessment of caspase-1 inhibitors.

Cas-1, as an inflammatory caspase, plays a crucial role in pyroptosis mediated by the canonical inflammasome pathway.^21^ Consequently, activated Cas-1 is regarded as a specific biomarker of pyroptosis. The characteristic of Cas-1, which enables the hydrolytic processing of various substrates involved in inflammatory signaling, suggests the presence of multiple peptide recognition motifs (Scheme 1a). Accordingly, a range of Cas-1-responsive imaging tools have been developed based on these substrate sequences. Most reported Cas-1 probes have been constructed by directly conjugating recognition peptides such as –YVAD– or –WEHD– to hemicyanine dyes or aggregation-induced emission (AIE) fluorogens.^22,23^ In contrast, relatively few Cas-1-responsive probes have been designed using fluorescence resonance energy transfer (FRET) strategies.^24,25^ With the growing understanding of the diverse biological roles of Cas-1 in pyroptosis and inflammation, a novel substrate motif –FLTD–, derived from the GSDMD cleavage site, has recently been identified.^26^ Despite its mechanistic relevance, only limited examples of fluorescent probes incorporating this sequence have been reported. For instance, Nandi *et al.* developed an inflammasome-activated theranostic nanoprobe integrating the –FLTD– sequence to achieve both Cas-1 imaging and drug delivery within a single nanoplatform.^27^ Inspired by these advances, we aimed to systematically evaluate how different Cas-1 recognition peptides affect probe performance when embedded within a unified FRET donor and quencher system. To this end, we designed a series of activatable Cas-1 probes incorporating three peptide sequences, namely –YVADG–, –WEHDG–, and –FLTDG–, and compared their detection behavior in terms of sensitivity, selectivity, and kinetic response. Among these candidates, the FLTDG-based probe FPy1, derived from the physiological substrate GSDMD, exhibited the most favorable sensing performance in aqueous solution, including a higher signal-to-background ratio and faster response rate (Scheme 1b). To validate its biological applicability, FPy1 and a reference probe YPy1 were evaluated in an NLRP3 inflammasome-mediated caspase-1 activation model. Confocal fluorescence imaging revealed that FPy1 displayed slightly higher signal contrast and responsiveness compared with YPy1, also confirming that FPy1 exhibits substantial promise for bioimaging applications. Building on these results, we further applied FPy1 to monitor caspase-1 activity and pyroptosis progression at multiple biological levels, including two-dimensional cell cultures, three-dimensional multicellular spheroids, and *in vivo* disease models, focusing on degenerative bone and joint diseases such as intervertebral disc degeneration and osteoarthritis (Scheme 1c). Moreover, leveraging the excellent sensitivity and stability of FPy1, we established a high-content screening platform for the discovery of small-molecule modulators of caspase-1 activity. Through this platform, deoxyshikonin was identified as a potential inhibitor that reduces Cas-1 expression and directly interacts with the enzyme to suppress its catalytic activity (Scheme 1c). Collectively, these results demonstrate that FPy1 is a versatile and specific fluorescent tool for tracking caspase-1 activation and pyroptosis progression, and further highlights FLTDG as a promising recognition motif for the future development of caspase-1 responsive probes.

## Results and discussion

### Design and synthesis of probes

**Fig. 1:**
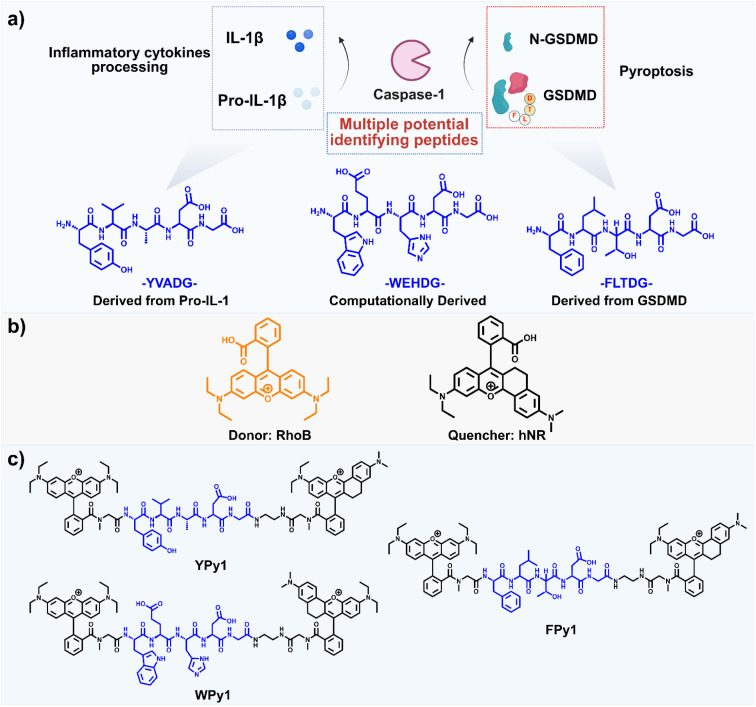
Probe structure for the rational design of Cas-1 fluorescent probe. (a) Potential recognition peptides inferred from the biological functions of caspase-1. (b) FRET system engineered with RhoB and hNR. (c) Structural architecture of engineered caspase-1 activatable probes.

Caspase-1 plays critical roles in inflammatory pathology by executing diverse biological functions, including the proteolytic processing of IL-1, IL-18, and pyroptosis-associated protein GSDMD.^28^ Therefore, there may be multiple potential recognition peptide sequences for caspase-1. Based on previous work,^23^ the peptide sequences –YVADG– and –WEHDG– are well-established caspase-1 recognition motifs, which have been widely utilized in the design of caspase-1 probes. Additionally, the peptide segment –FLTDG– derived from the pyroptosis executioner protein GSDMD has gained significant traction and offers a novel paradigm for designing the next-generation of caspase-1 probes (Fig. 1a). The FRET mechanism is a widely-used strategy in probe design, particularly for detecting proteases or other cleavage systems. The basis of FRET is the non-radiative energy transfer process between donors and acceptors.^29^ To achieve effective fluorescence resonance energy transfer (FRET) in caspase-1 activatable probes, the spectral overlap between the donor emission and the acceptor absorption is of crucial importance. Therefore, selecting an appropriate donor–acceptor pair is essential for constructing an efficient FRET system. To ensure efficient energy transfer and reliable fluorescence response, the photophysical properties of potential donor and acceptor candidates were carefully evaluated. The xanthene derivative hNR was found to exhibit a broad ultraviolet absorption band and a low fluorescence quantum yield. Owing to these characteristics, together with its facile synthesis, hNR was chosen as the quencher. Considering the absorption profile of hNR, rhodamine B, a well-established fluorophore with strong emission in the corresponding region, was selected as the donor to construct an efficient FRET system (Fig. S1a). Therefore, based on the aforementioned responsive peptides and the donor–quencher pairs, multiple caspase-1-responsive probes were constructed. The synthesis of the probes primarily involved three steps (Schemes S1–3). First, to effectively connect the donor and acceptor at both ends of the peptide chain, the structure of RhoB and hNR were respectively modified. RhoB-Sar and hNR-NH_2_ were synthesized through different synthetic pathways (Scheme S1). The intermediates, as well as RhoB-Sar and hNR were characterized using nuclear magnetic resonance (^1^H, ^13^C NMR) and high-resolution mass spectrometry (ESI-HRMS) (Fig. S22–S36). Subsequently, three peptides with the donor modified at the N-terminus were obtained through solid-phase synthesis (Scheme S2). Rho-FLTDG, Rho-YVADG and Rho-WEHDG were all characterized by ESI-HRMS (Fig. S37–39). Finally, the peptide fragments underwent amide condensation reactions with hNR-NH_2_ followed by side-chain deprotection to obtain the corresponding probes (Scheme S3). FPy1, YPy1 and WPy1 were all characterized by ESI-HRMS (Fig. S40–S42), LC-MSD was also employed to analyze the purity of individual probes, and the integrated peak results demonstrated that all probes had a purity of exceeding 90% (Fig. S43).

### Comprehensive analysis of the detection performance *in vitro*

**Fig. 2:**
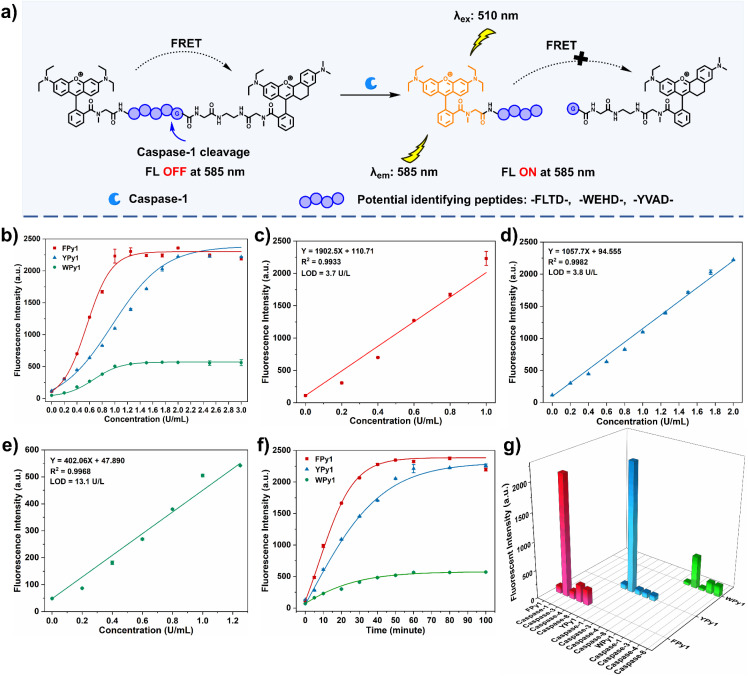
Comprehensive comparative analysis of the fluorescence response of probes to Cas-1. (a) Proposed mechanism of the fluorescence response of probes triggered by Cas-1. (b) The correlation between the fluorescence intensity at 585 nm of the probes and the Cas-1 concentration (0–3 U mL^−1^). (c) The linear fitting curve between the fluorescence intensity of FPy1 and Cas-1 concentration (0–1 U mL^−1^). (d) The linear fitting curve between the fluorescence intensity of YPy1 and Cas-1 concentration (0–2 U mL^−1^). (e) The linear fitting curve between the fluorescence intensity of WPy1 and Cas-1 concentration (0–1.25 U mL^−1^). (f) Time-dependent fluorescence response of the three probes treated with Cas-1 (2 U mL^−1^). (g) Fluorescence response of the three probes to caspase-3 (1 μg mL^−1^), caspase-4 (2 U mL^−1^), caspase-8 (1 μg mL^−1^) after incubation at 37 °C for 2 h. *λ*_ex_ = 510 nm, *λ*_em_ = 585 nm; photomultiplier voltage 700 V; slit widths 10.0 nm × 5.0 nm.

The spectral and chemical properties of all probes were then evaluated. Upon interaction with Cas-1, the specific peptide of the probes is cleaved, leading to the disruption of the FRET process in the system and the recovery of donor fluorescence (Fig. 2a). Prior to fluorescence detection, the UV-vis absorption spectra of the probes were acquired. The results indicated that the characteristic absorption peaks of RhoB and hNR were present (Fig. S1b–d). Subsequently, the sensitivity of the probes towards Cas-1 was measured in HEPES buffer (50 mM HEPES, 50 mM NaCl, 0.1% chaps, 5% glycerol, 10 mM DTT, 10 mM EDTA). The results indicated that the fluorescence intensity at 585 nm of all probes (5.0 μM) increased with concentrations of Cas-1. FPy1 and YPy1 exhibited similar response to Cas-1, with approximately 20.2-fold and 19.2-fold enhancement respectively, whereas WPy1 only displayed about 11.5-fold enhancement (Fig. S2a–c). The response differences among the probes were more intuitively observed by analyzing the standard curves of fluorescence intensity at 585 nm against the concentration of Cas-1 (Fig. 2b). Although WPy1 exhibited lower baseline fluorescence intensity at 585 nm, its maximum fluorescence intensity was significantly lower than that of FPy1 and YPy1, accounting for its reduced response. Furthermore, before reaching the maximum fluorescence intensity, FPy1 exhibited stronger fluorescence intensity at 585 nm compared to YPy1 at the same Cas-1 concentration. This result suggested that Cas-1 exhibited higher catalytic efficiency for FPy1 than YPy1. Due to the differences in sensitivity, the three probes demonstrated linear correlations with different concentration ranges of Cas-1, thus the limit of detection for the three probes were also different, with the lowest detection limit for FPy1, estimated to be 3.7 U L^−1^ (Fig. 2c–e). The reaction kinetics of the probes were also measured by monitoring the fluorescence intensity of FPy1, YPy1 and WPy1 (0.5–4 μM) following incubation with Cas-1 (2 U mL^−1^) (Fig. S3a–f). The reaction kinetics results indicated that FPy1 possessed the smallest Michaelis constant (KM), with a value of 4.42 μM. The reaction time of the probes was also investigated by monitoring the fluorescence spectra of the probes (5 μM) incubated with Cas-1 (2 U mL^−1^) at various time intervals (Fig. S4a–c). Analysis of the fluorescence change at 585 nm revealed that FPy1 exhibited the shortest reaction time, reaching maximum fluorescence intensity within approximately 40 min, whereas YPy1 and WPy1 required 60 min to reach maximum intensity (Fig. 2f). These results further corroborated the higher catalytic efficiency of Cas-1 for FPy1. Finally, the selectivity of the probes was evaluated. A series of potential biologically relevant interferents were selected, and the results illustrated that these interferents had no effect on the probes (Fig. S5a–c). Further selectivity validation was performed through incubating with caspase-3 (1 μg mL^−1^), caspase-4 (2 U mL^−1^), caspase-8 (1 μg mL^−1^) from the caspase protease family. It was found that, with the exception of WPy1, which exhibited a certain level of response to caspase-8, FPy1 and YPy1 exhibited almost no response to other caspases (Fig. 2g). Upon comprehensive analysis of the detection performance in solution, FPy1 exhibited relative advantages in sensitivity, reaction time, selectivity and reaction kinetics compared with YPy1 and WPy1. Therefore, FPy1 appeared to be the prime candidate for Cas-1 imaging applications. However, given that FPy1 and YPy1 exhibited similar performance in terms of their signal-to-background ratio for Cas-1 in aqueous solution, we further selected YPy1 as a reference probe for subsequent comparison of the intracellular imaging performance differences between FPy1 and YPy1.

### Fluorescence imaging of Cas-1 in THP-1 cells

**Fig. 3:**
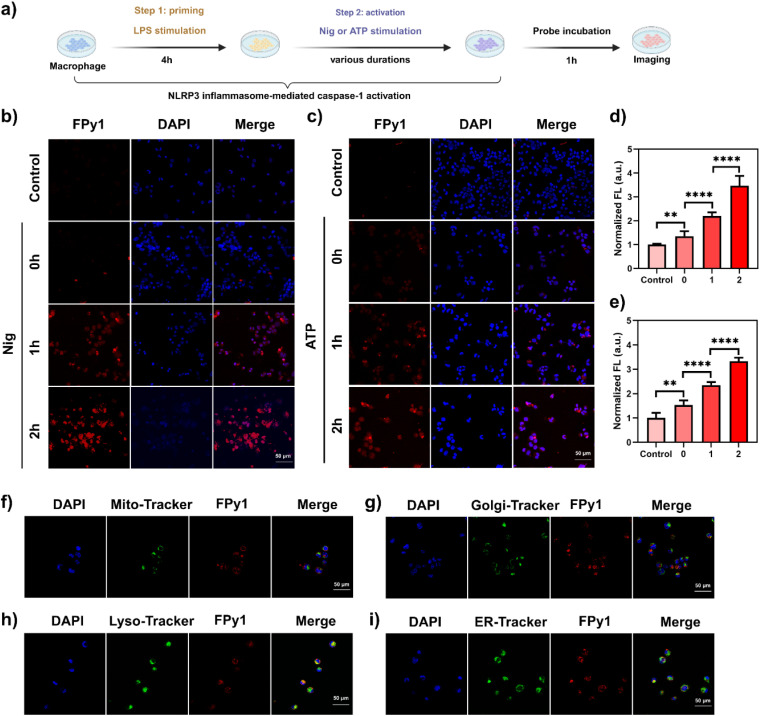
Bioimaging of Cas-1 in THP-1 cells stained with FPy1. (a) Illustration of fluorescence imaging in THP-1 cells with Cas-1 activation medicated by the NLRP3 inflammasome. (b) Confocal fluorescence images of THP-1 cells stained with FPy1 (5 μM). First row, control group; second row, LPS-treated cells were not stimulated with Nig; third row, LPS-treated cells were stimulated with Nig (10 μM) for 1 h; fourth row, LPS-treated cells were stimulated with Nig (10 μM) for 2 h. (c) Confocal fluorescence images of THP-1 cells stained with FPy1 (5 μM). First row, control group; second row, LPS-treated cells were not stimulated with ATP; third row, LPS-treated cells were stimulated with ATP (2 mM) for 1 h; fourth row, LPS-treated cells were stimulated with ATP (2 mM) for 2 h. (d) Quantitative analysis of fluorescence intensity in (b). (e) Quantitative analysis of fluorescence intensity in (c). (f) Fluorescence images of THP-1 cells treated with LPS (1 μg mL^−1^) and Nig (10 μM), followed by co-staining with FPy1 (5 μM, *λ*_ex_ = 514 nm, collected 530–610 nm), Mito-Tracker Green (50 μM, *λ*_ex_ = 488 nm, collected 495–525 nm) and DAPI (1 μg mL^−1^, *λ*_ex_ = 405 nm, collected 420–470 nm). (g) Fluorescence images of THP-1 cells treated with LPS (1 μg mL^−1^) and Nig (10 μM), followed by co-staining with FPy1 (5 μM, *λ*_ex_ = 514 nm, collected 530–610 nm), Golgi-Tracker Green (50 μM, *λ*_ex_ = 488 nm, collected 495–525 nm) and DAPI (1 μg mL^−1^, *λ*_ex_ = 405 nm, collected 420–470 nm). (h) Fluorescence images of THP-1 cells treated with LPS (1 μg mL^−1^) and Nig (10 μM), followed by co-staining with FPy1 (5 μM, *λ*_ex_ = 514 nm, collected 530–610 nm), Lyso-Tracker Green (50 μM, *λ*_ex_ = 488 nm, collected 495–525 nm) and DAPI (1 μg mL^−1^, *λ*_ex_ = 405 nm, collected 420–470 nm). (i) Fluorescence images of THP-1 cells treated with LPS (1 μg mL^−1^) and Nig (10 μM), followed by co-staining with FPy1 (5 μM, *λ*_ex_ = 514 nm, collected 530–610 nm), ER-Tracker Green (50 μM, *λ*_ex_ = 488 nm, collected 495–525 nm) and DAPI (1 μg mL^−1^, *λ*_ex_ = 405 nm, collected 420–470 nm). The corresponding Pearson's correlation for mitochondria, Golgi apparatus, lysosomes and endoplasmic reticulum were 0.4954, 0.4320, 0.5682, 0.4448, respectively. Statistical analyses were performed with one-way ANOVA with multiple comparisons, the significance levels were donated as ns (not significant), *P < 0.05, **P < 0.01, ***P < 0.001, and ****P < 0.0001.

Prior to fluorescence imaging experiments, the low cytotoxicity of FPy1 and YPy1 towards THP-1 cells was confirmed *via* CCK-8 assay (Fig. S6a and b). To assess the capability of FPy1 and YPy1 to monitor endogenous Cas-1, the classic NLRP3 inflammasome-mediated Cas-1 activation model was established in THP-1 cells. THP-1 cells were initially stimulated with LPS (1 μg mL^−1^) for 4 h to prime the production of NLRP3 inflammasome-associated proteins. Subsequently, the cells were stimulated with Nigericin (Nig, 10 μM) or ATP (2 mM) for various durations to induce the activation of Cas-1 and the pyroptosis-related pathway. The THP-1 cells were further stained with FPy1 or Ypy1 (5 μM) for 1 h and imaged (Fig. 3a). The imaging results indicated that the fluorescence intensity of both FPy1 and YPy1 increased with prolonged stimulation time by Nig or ATP (Fig. 3b–c, S7a and b). Quantitative-analysis results of intracellular fluorescence further demonstrated that the fluorescence intensity increased with the duration of Nig or ATP stimulation (Fig. 3d, e, S7c and d). Western blot (WB) analysis confirmed that, regardless of whether Nig or ATP was used as the stimulant, the level of activated Cas-1 was consistent with the fluorescence imaging results (Fig. S8a–d). However, quantitative confocal fluorescence analysis revealed that under 2 h of Nig or ATP stimulation, the fluorescence increase of FPy1 was slightly higher than that of YPy1. Therefore, FPy1 may be a more suitable candidate for monitoring low caspase-1 levels in subsequent imaging studies. We further employed flow cytometry to evaluate changes in fluorescence intensity of the FPy1 when THP-1 cells were stimulated with Nig or ATP, and the results were consistent with the above imaging findings (Fig. S9a–d). To investigate the distribution of FPy1 within cells, THP-1 cells were co-stained with FPy1 and commercially available organelle-tracking dyes, including Mito-Tracker Green (mitochondria), Golgi-Tracker Green (Golgi apparatus), Lyso-Tracker Green (lysosomes) and ER-Tracker Green (endoplasmic reticulum). All dyes were imaged in the green fluorescence channel to avoid overlap with the red emission of FPy1 (Fig. 3f–i). The fluorescence imaging results indicated that the red fluorescence of FPy1 exhibited stronger colocalization with the green fluorescence of Lyso-Tracker Green than with other organelle markers. The corresponding Pearson's correlation coefficients for mitochondria, Golgi apparatus, lysosomes, and endoplasmic reticulum were 0.4954, 0.4320, 0.5682, and 0.4448, respectively. These results suggested that while FPy1 may possess partial lysosomal targeting capability, it predominantly exhibited a diffuse intracellular distribution. To further confirm the specificity of FPy1 for Cas-1 imaging in living cells, THP-1 cells with Cas-1 knockdown *via* siRNA were constructed. WB analysis confirmed that the activated Cas-1 in the Si-Casp1 group was significantly lower than that in the Si-Scramble group, indicating the successful knockdown of Cas-1 in Si-Casp1 group (Fig. S10a and b). As shown in Fig. S11a, even when the THP-1 cells in the Si-Casp1 group were stimulated by LPS and Nig, the intracellular fluorescence intensity was significantly lower than that in the Si-Scramble group. Fluorescence quantitative analysis revealed significant differences in fluorescence intensity between the Si-Casp1 group and the Si-Scramble group (Fig. S11b). Flow cytometric results also indicated that the fluorescence intensity in the Si-Casp1 group was lower than that in the Si-Scramble group (Fig. S11c and d). These findings indicated that FPy1 can be effectively and specifically employed to monitor activated Cas-1 in living cells.

### Multi-scale fluorescence imaging of Cas-1 in intervertebral disc degeneration

**Fig. 4:**
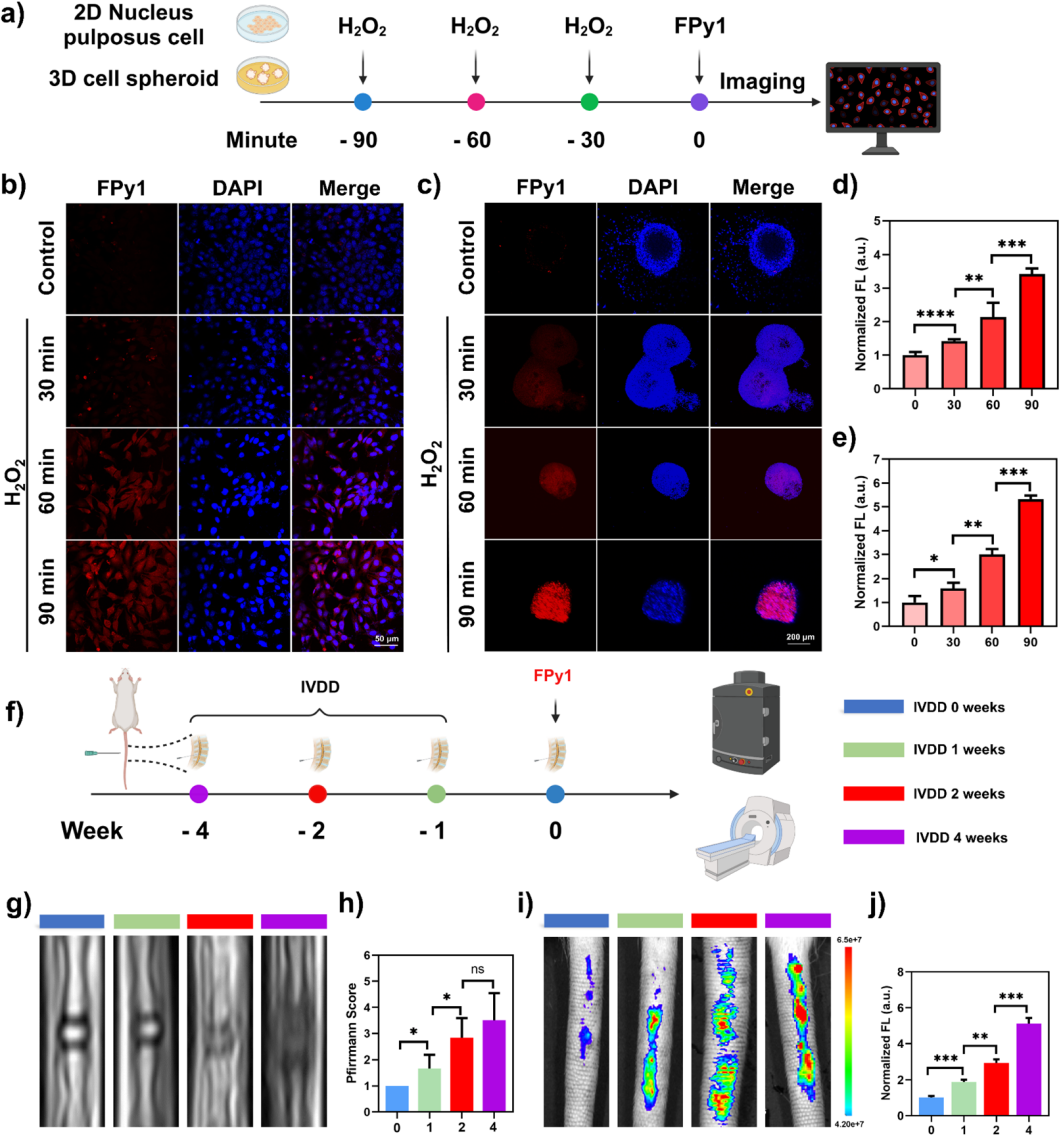
Multi-scale bioimaging of Cas-1 in primary nucleus pulposus cells, cell spheroids and *in vivo* stained with FPy1. (a) The illustration of fluorescence imaging in NP cells and spheroids stimulated by H_2_O_2_ (2 mM). (b) Confocal fluorescence images of NP cells stained with FPy1 (5 μM). First row, control group; second row, NP cells were stimulated with H_2_O_2_ for 30 min; third row, NP cells were stimulated with H_2_O_2_ for 60 min; fourth row, NP cells were stimulated with H_2_O_2_ for 90 min. FPy1 (*λ*_ex_ = 514 nm, collected 530–610 nm); DAPI (*λ*_ex_ = 405 nm, collected 420–470 nm). (c) Confocal fluorescence images of cell spheroids stained with FPy1 (5 μM). First row, control group; second row, spheroids were stimulated with H_2_O_2_ for 30 min; third row, spheroids were stimulated with H_2_O_2_ for 60 min; fourth row, spheroids were stimulated with H_2_O_2_ for 90 min. FPy1 (*λ*_ex_ = 514 nm, collected 530–610 nm); DAPI (*λ*_ex_ = 405 nm, collected 420–470 nm). (d) Quantitative analysis of fluorescence intensity in (b). (e) Quantitative analysis of fluorescence intensity in (c). (f) Scheme of fluorescence imaging or MRI in puncture-induced IVDD rat model. (g) Representative MRI images of intervertebral discs in rats at 0, 1, 2 and 4 weeks after puncture. (h) Pfirrmann grade from *T*_2_-weighted MRI in each group. (i) Fluorescence images of rat intervertebral discs at 0, 1, 2 and 4 weeks after puncture. (j) Quantitative analysis of fluorescence intensity in (i). Statistical analyses were performed with one-way ANOVA with multiple comparisons, the significance levels were donated as ns (not significant), *P < 0.05, **P < 0.01, ***P < 0.001, and ****P < 0.0001.

With advancing age, the prevalence of degenerative bone and joint disorders (DBJDs), such as intervertebral disc degeneration (IVDD) and osteoarthritis (OA), increases markedly among middle-aged and elderly individuals.^30^ Chronic inflammation has been identified as the underlying cause of DBJDs. In particular, inflammasome-mediated pyroptosis has been recognized as a primary contributor to sustained inflammatory responses.^31^ Consequently, pyroptosis-associated biomarkers represent promising indicators for the diagnosis and monitoring of DBJDs. IVDD, a chronic degenerative condition closely related to natural aging, has become one of the leading causes of disability worldwide.^32^ In recent years, numerous studies have highlighted the critical involvement of NLRP3 inflammasome-mediated pyroptosis in the pathogenesis of IVDD.^33^ Previous studies have demonstrated that excessive reactive oxygen species can significantly disrupt the homeostasis of the intervertebral disc (IVD) microenvironment, leading to pyroptosis of nucleus pulposus cells (NP cells).^34,35^ Thus, H_2_O_2_ was selected to stimulate rat primary NP cells for various durations and induce IVDD. H_2_O_2_-treated NP cells were stained with FPy1 (5 μM) and imaged (Fig. 4a). The imaging results indicated a positive correlation between fluorescence intensity and H_2_O_2_-stimulation time (Fig. 4b). The fluorescence quantitative analysis also intuitively demonstrated that the intracellular fluorescence intensity increased with the duration of H_2_O_2_ stimulation (Fig. 4d). The WB results confirmed that the level of activated Cas-1 gradually increased under H_2_O_2_ exposure (Fig. S12). To better simulate the tissue-like microenvironment, NP cells were further cultured as cell spheroids. The cell spheroids were stimulated with H_2_O_2_ (2 mM) for varying durations, followed by FPy1 staining and imaging. The results of fluorescence imaging in spheroids and corresponding quantitative analysis were consistent with the trends of fluorescence changes observed in 2D cells (Fig. 4c and e), but the fluorescence enhancement in the spheroids was more pronounced. Encouraged by the promising imaging results of FPy1 in NP cells and cell spheroids, we further assessed its capability for imaging pytoptosis *in vivo*. Intervertebral discs between coccyx vertebra 5 and 6, 6 and 7, and 7 and 8 (Co5/6, Co6/7, Co7/8) were identified and subjected to needle punctures for varying durations to establish a series of IVDD models in rats (Fig. 4f). *T*_2_-weighted MRI was used to assess the degree of IVDD at various stages. Healthy IVD typically appear bright white on *T*_2_-weighted MRI, while degenerated IVD are gray or black.^36^ As shown in Fig. 4g, the *T*_2_-weighted MRI signal from the IVD gradually diminished as the duration of needle puncture increased. Pfirrmann scoring results from *T*_2_-weighted MRI reflected the progression of disc degeneration (Fig. 4h); however, it was noted that the Pfirrmann scoring did not significantly distinguish between 2-weeks and 4-weeks IVDD. Following intradiscal injection of FPy1, the fluorescence images of the tail modeling site were obtained from the dorsal aspect. As shown in Fig. 4i, puncture led to a significant enhancement of fluorescence at the corresponding areas. Quantitative analysis indicated that FPy1 could significantly differentiate the four different stages of IVDD based on fluorescence intensity (Fig. 4j), potentially aiding Pfirrmann scoring and more accurately classifying IVDD stages. Histological analysis was also conducted to evaluate tissue alterations across various groups. Hematoxylin-eosin staining demonstrated that IVD for the non-puncture group maintained intact structure, with a clear demarcation between the annulus fibrosus (AF) and NP tissues (Fig. S13a). In contrast, puncture treatment led to structural destruction of the IVD, such as the loss of NP tissue and reduction of the boundary between NP and AF tissues. Safranin O-fast green staining further revealed that puncture caused severe tissue fibrosis, and extracellular matrix loss within the IVD (Fig. S13a). Histological scoring result corroborated these findings, reflecting the same trends as with fluorescence imaging observed using FPy1 (Fig. S13b). Immunofluorescence staining was used to detect activated Cas-1 and its downstream pyroptosis-related protein, N-GSDMD, in the NP region of the rat tail. The immunofluorescence results indicated that the levels of activated Cas-1 and N-GSDMD both gradually increased with the duration of puncture, consistent with the *in vivo* fluorescence changes observed using FPy1 (Fig. S14a–c). Additionally, immunohistochemical (IHC) staining results of NLRP3 and IL-1 at the model sites revealed that all the aforementioned pyroptosis-related inflammatory markers showed an increasing trend with prolonged modeling duration (Fig. S15). Given the imaging performance described above, FPy1 was capable of enabling Cas-1 and pyroptosis imaging across multi-scales of intervertebral disc degeneration-related biological models.

### Multi-scale fluorescence imaging of Cas-1 in osteoarthritis

**Fig. 5:**
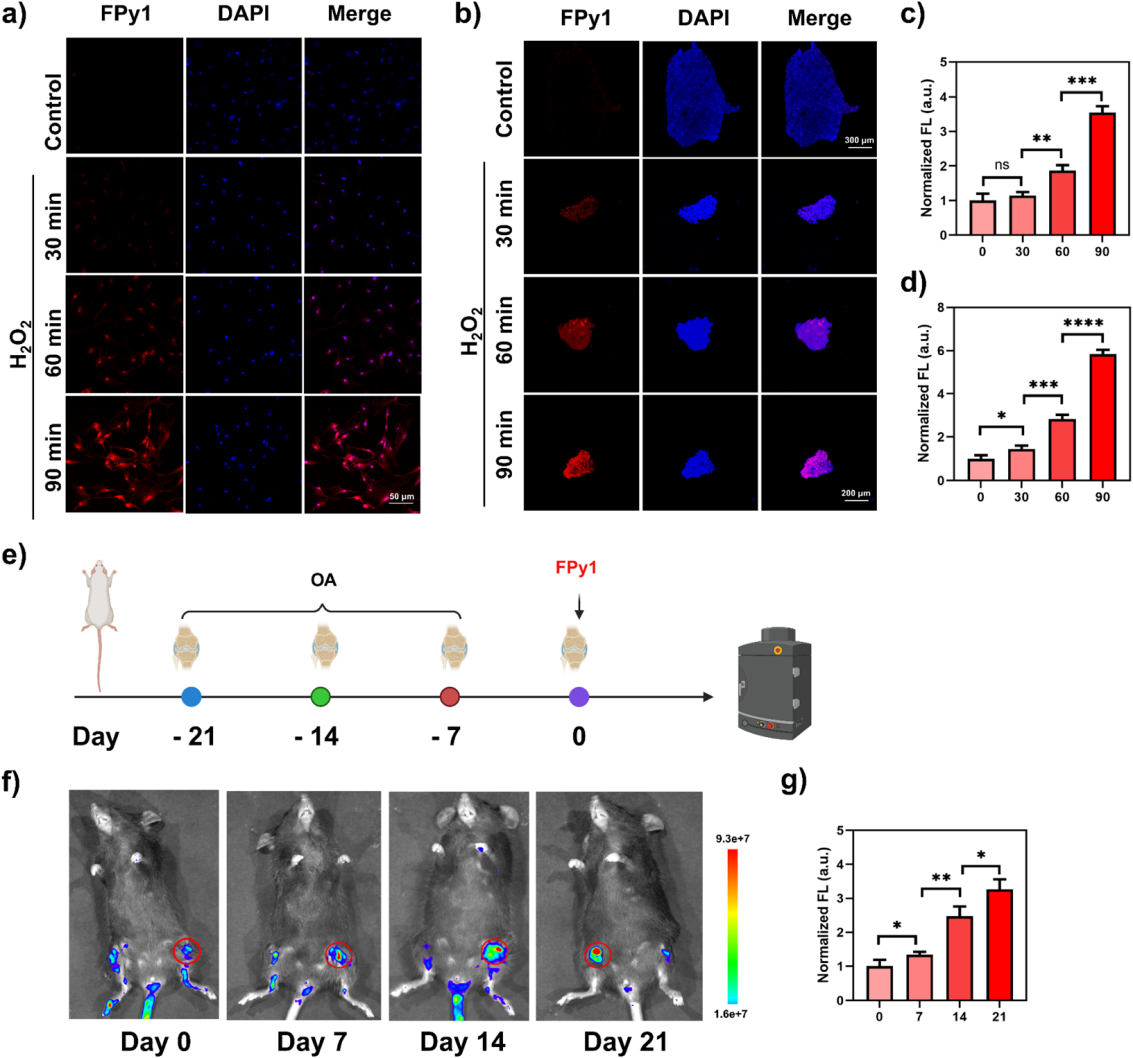
Fluorescence imaging of FPy1 in osteoarthritis-related biological models. (a) Confocal fluorescence images of primary chondrocytes stained with FPy1 (5 μM). Top row, control group; second row, chondrocytes stimulated with H_2_O_2_ (2 mM) for 30 min; third row, chondrocytes stimulated with H_2_O_2_ (2 mM) for 60 min; bottom row, chondrocytes stimulated with H_2_O_2_ (2 mM) for 90 min. FPy1 (*λ*_ex_ = 514 nm, collected 530–610 nm); DAPI (*λ*_ex_ = 405 nm, collected 420–470 nm). (b) Confocal fluorescence images of cell spheroids stained with FPy1 (5 μM). Top row, control group; second row, spheroids stimulated with H_2_O_2_ (2 mM) for 30 min; third row, spheroids stimulated with H_2_O_2_ (2 mM) for 60 min; bottom row, spheroids were stimulated with H_2_O_2_ (2 mM) for 90 min. FPy1 (*λ*_ex_ = 514 nm, collected 530–610 nm); DAPI (*λ*_ex_ = 405 nm, collected 420–470 nm). (c) Quantitative analysis of fluorescence intensity in (a). (d) Quantitative analysis of fluorescence intensity in (b). (e) Schematic illustration of osteoarthritis modeling and fluorescence imaging. (f) Fluorescence images of mouse knee joints acquired on days 0, 7, 14, and 21 post-modeling. (g) Quantitative analysis of fluorescence intensity in (f). Statistical analyses were performed with one-way ANOVA with multiple comparisons, the significance levels were donated as ns (not significant), *P < 0.05, **P < 0.01, ***P < 0.001, and ****P < 0.0001.

OA, another common DBJD, is primarily characterized by severe pain, joint stiffness, and reduced mobility, which significantly impair a patients' quality of life.^37^ The decline in chondrocyte function plays a crucial role in the pathogenesis of OA. Inflammasome-mediated chondrocyte pyroptosis has been closely associated with matrix degradation and cartilage degeneration in OA.^38^ To model OA *in vitro*, primary chondrocytes were stimulated with H_2_O_2_ (2 mM) for different durations, followed by staining with FPy1 (5 μM) and fluorescence imaging to monitor the progression of pyroptosis under oxidative stress. Both fluorescence images and corresponding quantitative analysis confirmed that the intracellular fluorescence intensity of FPy1 in primary chondrocytes increased with prolonged H_2_O_2_ stimulation (Fig. 5a and c). The WB results further demonstrated that the level of activated Cas-1 gradually increased in chondrocytes upon H_2_O_2_ treatment (Fig. S16a and b). To better mimic the *in vivo* pathological features of OA, primary chondrocytes were also cultured as three-dimensional spheroids and stimulated with H_2_O_2_. FPy1 staining and fluorescence imaging revealed more pronounced fluorescence enhancement in chondrocyte spheroids than in two-dimensional cultures, consistent with the fluorescence patterns observed in NP spheroids (Fig. 5b and d). Furthermore, we evaluated the capability of FPy1 to monitor pyroptosis progression at different stages of OA in a mouse model. Micro-scalpel surgery was performed to transect the anterior cruciate ligament (ACL) of the knee, thereby inducing joint injury. OA models at distinct stages were obtained by maintaining the mice for 0, 7, 14, and 21 days post-surgery (Fig. 5e). As shown in Fig. 5f, the fluorescence signals from FPy1 in the knee joints of mice exhibited clear stage-dependent variations, with the intensity gradually increasing as the modeling duration was extended (Fig. 5g). Safranin O-fast green staining further revealed severe fibrosis and extracellular matrix loss in the knee joints of the model mice (Fig. S17). The pathological manifestations in the corresponding tissues became more pronounced with longer modeling periods, consistent with the fluorescence imaging results obtained using FPy1. Moreover, immunohistochemical (IHC) staining of NLRP3 and IL-1 at the model sites showed an increasing expression trend with extended modeling duration (Fig. S18). Collectively, these findings further confirm that FPy1 can effectively monitor Cas-1 activation and pyroptosis progression across different biological contexts, including osteoarthritis.

### Constructing a high-content screening system based on FPy1 for the discovery of caspase-1 modulators

**Fig. 6:**
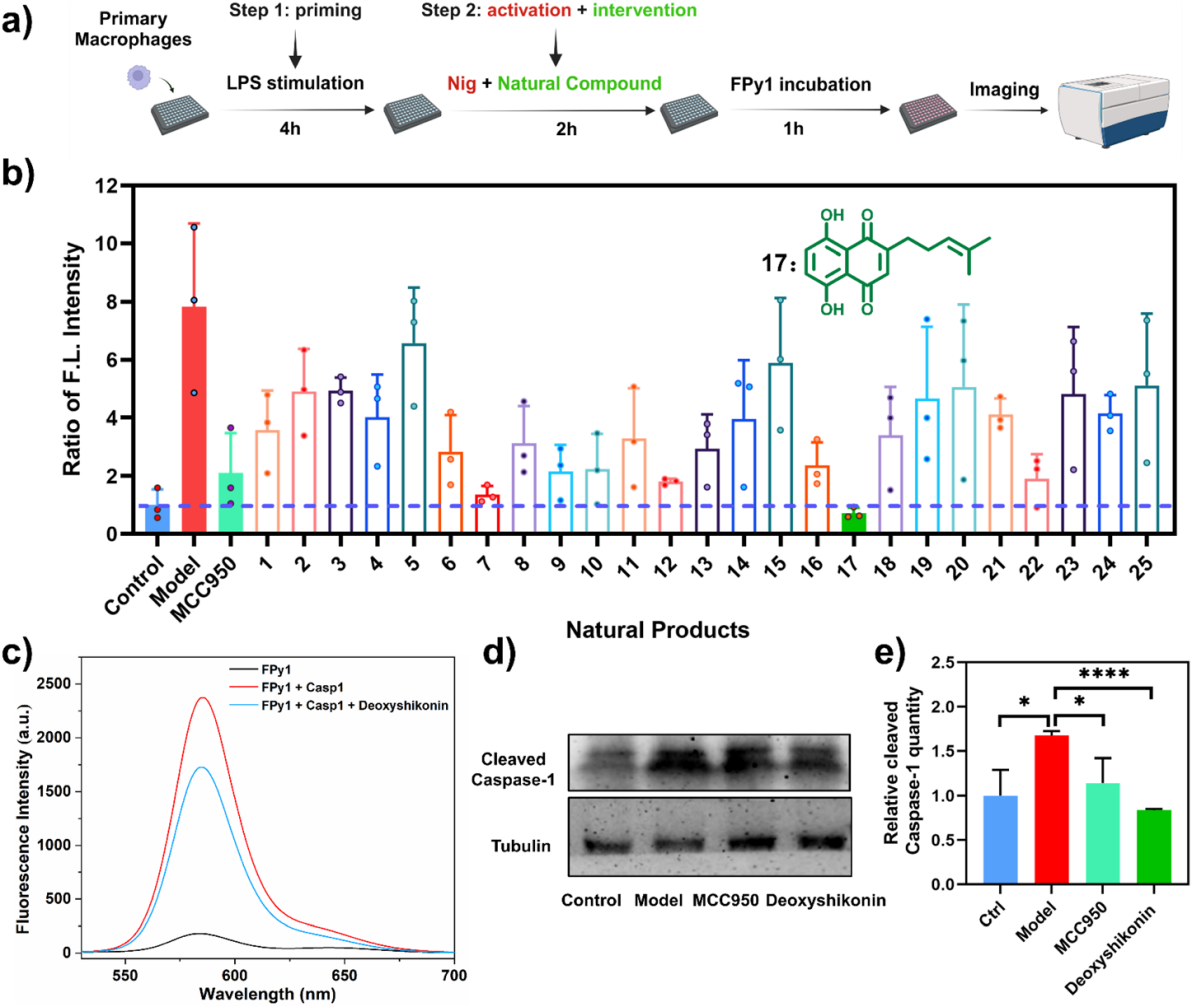
High-content screening for Cas-1 modulators from natural products by FPy1 staining. (a) Illustration of high-content screening workflow. (b) The relative ratio of fluorescence intensity under the intervention of natural products. The fluorescence intensity of the DMSO-treated group was set as 1. Inset: molecular structure of natural product 17. (c) Fluorescence intensity of FPy1 in the presence and absence of deoxyshikonin. (d) Cleaved Cas-1 in control and treated groups that were examined by WB. (e) The relative ratio of the grey level of cleaved Cas-1 in (d) analyzed by Image J. Statistical analyses were performed with one-way ANOVA with multiple comparisons, the significance levels were donated as ns (not significant), *P < 0.05, **P < 0.01, ***P < 0.001, and ****P < 0.0001.

The efficient and rapid discovery of Cas-1 modulators is of significant importance considering the therapeutic potential of Cas-1 in various diseases.^39,40^ Therefore, we employed FPy1 to construct a high-content screening system aimed at identifying Cas-1 modulators. The level of activated Cas-1 in cells is influenced by multiple factors. To reduce the impact of factors that affect Cas-1 activation in cells, we utilized the NLRP3 inflammasome-mediated Cas-1 activation model, administering the drug during the second stage where the zymogen was converted into activated Cas-1. MCC950, a selective NLRP3 inflammasome inhibitor that could suppress Cas-1 activation, was selected as the positive drug.^41^ Furthermore, the THP-1 cells were replaced by primary peritoneal macrophages (PMs), as PMs could exhibit more pronounced changes in activated Cas-1 levels under stimulation. To validate the efficacy of the Cas-1 modulator screening system (Fig. 6a), we first observed the fluorescence images of PMs stained with FPy1 (5 μM) in the control group, model group, and positive drug group. As shown in Fig. S19a, the model group exhibited a significant fluorescence enhancement compared to the control group. Conversely, the positive drug group, incubated with MCC950 (1 μM) and Nig (10 μM) simultaneously, demonstrated a marked reduction in fluorescence relative to the model group. Quantitative fluorescence analysis further confirmed that the fluorescence intensity in the positive drug group was significantly lower than in the model group (Fig. S19b). These results indicated that the screening system could effectively identify potential modulators based on fluorescence intensity. Subsequently, the impact of 25 natural products (10 μM) on activated Cas-1 were studied using high-content analysis (Fig. S20). The names and structures of all compounds are summarized in Fig. S21. Quantitative fluorescence analysis revealed that these natural products reduced fluorescence signals (Fig. 6b). Importantly, compound 17, deoxyshikonin, demonstrated a significant reduction in fluorescence intensity compared to the model group. To determine whether deoxyshikonin directly binds to Cas-1 and inhibits its activity, we conducted fluorescence detection following co-incubation of deoxyshikonin, FPy1 and Cas-1. The fluorescence spectrum indicated that the fluorescence at 585 nm was lower in the presence of deoxyshikonin, suggesting deoxyshikonin could directly inhibit Cas-1 (Fig. 6c). Furthermore, the WB results indicated that the deoxyshikonin could significantly inhibit the activation of Cas-1, reducing activated Cas-1 levels (Fig. 6d and e). Overall, FPy1 could serve as an effective tool for the rapid identification of lead compounds that modulate Cas-1 activity within a high-content analysis platform.

## Conclusions

In summary, using different recognition peptides of Cas-1, we developed a series of probes within the same fluorescence reporting system to investigate how peptide sequences influence probe performance. Comprehensive evaluation of the detection parameters in aqueous solution and imaging results in THP-1 cells revealed that FPy1 exhibited the best overall performance. The ability of FPy1 to specifically image endogenous Cas-1 in living cells was confirmed using a classic Cas-1 activation model in both normal and Cas-1 knockdown THP-1 cells. Furthermore, in degenerative bone and joint disease models, including intervertebral disc degeneration and osteoarthritis, FPy1 successfully enabled Cas-1 imaging and pyroptosis monitoring across three biological application scenarios: cells, cell spheroids, and *in vivo* models. Notably, we established a high-content screening platform for Cas-1 modulators using the classic activation model in primary macrophages. Among the identified modulators, deoxyshikonin, was shown to significantly reduce Cas-1 levels and inhibit activity by direct binding. Overall, FPy1 has been proven to be a versatile tool for monitoring Cas-1 activity and assessing pyroptosis across various application scenarios. Moreover, the peptide sequence –FLTDG– was shown to exhibit great potential as an excellent recognition motif for developing activatable Cas-1 probes. These findings provide valuable insights and a solid foundation for the rational design of next-generation Cas-1 activatable fluorescent probes and for advancing pyroptosis imaging research.

## Ethical statement

All animal experiments were performed in compliance with the relevant guidelines and regulations of the People's Republic of China for the care and use of experimental animals (Regulations on the Administration of Laboratory Animals of the People's Republic of China, *etc.*). All animal procedures were performed in accordance with the Guidelines for Care and Use of Laboratory Animals of Hangzhou Medical College and approved by the Animal Ethics Committee of Hangzhou Medical College (ethical approval number: ZJCLA-IACUC-20011003 and ZJCLA-IACUC-20011048).

## Author contributions

The manuscript was written through contributions of all authors. All authors have given approval to the final version of the manuscript.

## Conflicts of interest

There are no conflicts to declare.

## Supplementary Material

SC-017-D5SC07690K-s001

## Data Availability

The source data underlying this work will be made available by the corresponding author Yi Wang upon reasonable request. The data that support the findings of this study are available in the supplementary information (SI) of this article. Supplementary information is available. See DOI: https://doi.org/10.1039/d5sc07690k.
