# Supplementary material for: Design and optimization of caspase-1-responsive fluorescent probes for pyroptosis imaging and anti-pyroptosis drug screening
Source: Chem Sci. 2025 Nov 10;17(1):585–96. doi: 10.1039/d5sc07690k (PMC12598616; doi:10.1039/d5sc07690k)
Supplement: SC-017-D5SC07690K-s001 [file SC-017-D5SC07690K-s001.pdf]

Supplementary Information (SI) for Chemical Science.
This journal is © The Royal Society of Chemistry 2025

Supporting Information

Design and Optimization of Caspase-1-Responsive Fluorescent Probes for Pyroptosis Imaging and Anti-Pyroptosis Drug Screening

Wei Wang,[#ab] Guanrui Huang,[#c] Yeting Zhou,[ab] Yue Wang,[c] Luling Wu,[*de] Tony D. James,[eh] Weili Wang,[*f], Yi Wang[*abg]

[a] Pharmaceutical Informatics Institute, College of Pharmaceutical Sciences, Zhejiang University, Hangzhou 310058, China. E-mail: zjuwangyi@zju.edu.cn

[b] National Key Laboratory of Chinese Medicine Modernization, Innovation Center of Yangtze River Delta, Zhejiang University, Jiaxing 314102, China.

[c] Department of Orthopedic Surgery, the First Affiliated Hospital, Zhejiang University School of Medicine, Hangzhou 310003, China.

[d] State Key Laboratory of Analytical Chemistry for Life Science, School of Chemistry and Chemical Engineering, Nanjing University, 163 Xianlin Avenue, Nanjing, 210023, China. E-mail: lulingwu@nju.edu.cn

[e] Department of Chemistry, University of Bath, BA2 7AY, UK.

[f] Department of Hepatobiliary and Pancreatic Surgery, The Second Affiliated Hospital, Zhejiang University School of Medicine, Hangzhou, 310009, China. E-mail: wangweili@zju.edu.cn

[g] Jinan Microecological Biomedicine Shandong Laboratory, Jinan 250118, China

[h] School of Chemistry and Chemical Engineering, Henan Normal University, Xinxiang. 453007, China

[#] These authors contributed equally.

Materials and methods

General materials

The Fmoc-protected amino acid used for solid-phase peptide synthesis (SPPS) and the starting chemical regents were purchased from Sigma-Aldrich (Shanghai, China) and Bidepharm (Shanghai, China). All chemicals were of reagent grade. The resin used for SPPS was purchased from GL Biochem (Shanghai, China). Lipopolysaccharide (LPS, L2630), Nigericin and ATP (11140965001) were purchased from Merck (Shanghai, China) and Invitrogen (Shanghai, China), respectively. Cell culture plates were sourced from Wuxi NEST Biotechnology. Recombinant human caspase-1, caspase-3, caspase-4, and caspase-8 were obtained from Abcam and MCE (Shanghai, China). Anti-cleaved caspase-1 antibody, anti-GAPDH antibody, and goat anti-Rabbit/anti-mouse lgG/HRP were purchased from CST (USA) and Beyotime (Shanghai, China). Fetal bovine serum, DMEM, penicillin/streptomycin, RPMI 1640 medium were purchased from Thermo Fisher Scientific (Shanghai, China). Sprague-Dawley rats were purchased from the Animal Center at Hangzhou Medical College. At the animal facility at Hangzhou Medical College, rats were bred, housed and used under specific pathogen-free conditions. All animal studies were performed with the approval of the ethical review board of Hangzhou Medical College (ethical approval number ZJCLA-IACUC-20011003).

Instruments

The ^{1}H NMR and ^{13}C NMR spectra were recorded using a 500 MHz Bruker AV 500 spectrometer. UV-vis measurements were performed on a Tecan Infinite M1000 Pro Multi-function microplate reader, and fluorescence spectra were acquired using a Hitachi F-2700 spectrophotometer. Mass spectra were obtained using an Agilent 6546 instrument. Fluorescent images of cells, cell spheroid and tissue slices were captured with an Olympus IX83-FV3000 microscope.

Synthesis and characterization

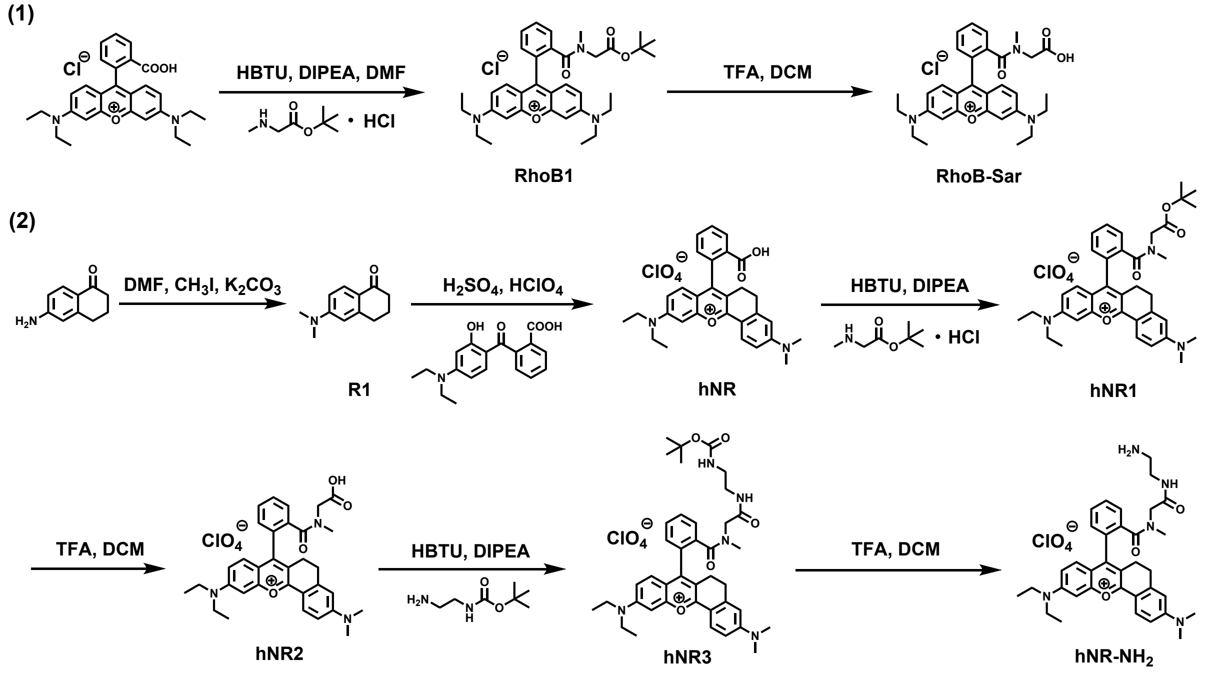

Scheme S1. Donor-Acceptor structural modification.

Synthesis of RhoB1

Rhodamine B (479 mg, 1 mmol) and HBTU (379 mg, 1 mmol) were dissolved in DMF (10 mL), followed by the addition of DIPEA (495 μL, 3 mmol). The resulting mixture was stirred for 0.5 hours. Subsequently, tert-butyl sarcosinate hydrochloride (272 mg, 1.5 mmol) was added, and the reaction mixture was stirred continuously for 24 hours. Upon completion of the reaction, the mixture was poured into deionized H_2O (50 mL), and then the precipitate was filtered and washed with water. The crude compound, RhoB1, was obtained as pink solid without further purification (373 mg, 62 % yield). ^{1}H NMR (500 MHz, DMSO-d_6) δ 7.77 – 7.74 (m, 2H), 7.64 (dd, J = 7.1, 1.7 Hz, 1H), 7.54 (dd, J = 6.5, 2.3 Hz, 1H), 7.16 (1H), 7.14 (1H), 7.09 (d, J = 2.3 Hz, 1H), 7.07 (d, J = 2.4 Hz, 1H), 6.93 (d, J = 2.3 Hz, 2H), 3.78 (s, 2H), 3.67-3.61 (m, 8H), 2.80 (s, 3H), 1.23 (s, 9H), 1.20 (t, J = 7.0 Hz, 12H). ^{13}C NMR (126 MHz, DMSO-d_6) δ 168.21, 167.21, 157.32, 155.14, 154.89, 135.48, 131.96, 130.19, 130.09, 129.90, 129.49, 127.07, 113.92, 113.36, 95.60, 80.66, 48.88, 45.25, 38.11, 27.44, 12.39. HRMS (ESI$^+$): calcd for $C_{35}H_{44}N_3O_4^+$ 570.3326 m/z, found, 570.3342 m/z.

Synthesis of RhoB-Sar

RhoB1 (303 mg, 0.5 mmol) was dissolved in anhydrous DCM (10 mL), followed by the addition of TFA (1mL). The solution was stirred at room temperature for 3 hours. Afterward, the solvent was removed under vacuum, and the residue was purified by silica gel column chromatography (DCM/MeOH, 150/1) to afford RhoB-Sar as a pink solid (230 mg, 84 % yield). ^{1}H NMR (500 MHz, DMSO-d_6) δ 7.76 – 7.62 (m, 4H), 7.51-7.46 (m, 1H), 7.13 (d, J = 3.5 Hz, 2H), 7.09 (dd, J = 9.7, 1.9 Hz, 1H), 6.93-6.90 (m, 2H), 3.75 (s, 2H), 3.66-3.63 (m, 8H), 2.80 (s, 3H), 1.20 (t, J = 6.7 Hz, 12H). HRMS (ESI$^+$) calcd for $C_{31}H_{36}N_3O_4^+$ 514.2700 m/z, found, 514.2715 m/z.

Synthesis of R1

6-Amino-3,4-dihydro-1(2H)-naphthalenone (1.3 g, 8 mmol) and K_2CO_3 (3.3 g, 24 mmol) were dissolved in DMF (10 mL), followed by the addition of CH_3I (2.8 g, 20 mmol). The mixture was stirred at 45 °C for 24 hours. The reaction solution was then extracted with ethyl acetate (EA), and the organic layers were dried over Na_2SO_4. After removing EA under vacuum, the crude product was purified by silica gel column chromatography (PE/EA, 20/1) to yield R1 as

colorless crystals (1.1 g, 73 % yield). ^{1}H NMR (500 MHz, DMSO-d_6) δ 7.70 (d, J = 8.9 Hz, 1H), 6.63 (dd, J = 8.9, 2.6 Hz, 1H), 6.48 (d, J = 2.6 Hz, 1H), 3.00 (s, 6H), 2.82 (t, J = 6.0 Hz, 2H), 2.43 (t, J = 6.4 Hz, 2H), 1.98 – 1.93 (m, 2H). ^{13}C NMR (126 MHz, DMSO-d_6) δ 195.43, 153.40, 146.39, 128.45, 121.00, 110.18, 109.48, 39.68, 38.46, 29.85, 23.27. HRMS (ESI$^+$): calcd for $C_{12}H_{16}NO$ [M+H]$^+$: 190.1226 m/z, found, 190.1230 m/z.

Synthesis of hNR

2-(4-Diethylamino-2-hydroxybenzoyl)benzoic acid (627 mg, 2 mmol) and R1 (378 mg, 2 mmol) were dissolved in H_2SO_4 (10 mL) and the reaction mixture was stirred at 90 °C for 3 hours. After cooling to room temperature, the solution was poured into ice water, and $HClO_4$ (1.5 mL) was added. The precipitate was filtered, and the crude product was purified by silica gel column chromatography (DCM/MeOH, 60/1) to obtain hNR as a dark purple solid (807 mg, 71 % yield). ^{1}H NMR (500 MHz, DMSO-d_6) δ 13.2 (s, 1H) 8.21 – 8.17 (m, 2H), 7.86 (ddd, J = 7.6, 7.6, 1.3 Hz, 1H), 7.76 (ddd, J = 7.7, 7.7, 1.2 Hz, 1H), 7.41 (dd, J = 7.6, 1.3 Hz, 1H), 7.23 (d, J = 2.5 Hz, 1H), 7.08 (dd, J = 9.4, 2.5 Hz, 1H), 6.92 (dd, J = 9.2, 2.5 Hz, 1H), 6.87 (d, J = 9.4 Hz, 1H), 6.74 (d, J = 2.4 Hz, 1H), 3.58 (q, J = 7.2 Hz, 4H), 3.18 (s, 6H), 2.95 – 2.88 (m, 1H), 2.85 – 2.79 (m, 1H), 2.54 – 2.40 (m, 2H), 1.20 (t, J = 7.0 Hz, 6H). ^{13}C NMR (126 MHz, DMSO-d_6) δ 166.44, 163.36, 156.12, 155.02, 153.04, 145.04, 134.32, 132.95, 130.85, 129.93, 129.18, 129.03, 128.58, 118.38, 115.18, 114.27, 112.93, 111.98, 110.68, 95.97, 54.86, 44.77, 39.94, 26.84, 23.54, 12.36. HRMS (ESI$^+$): calcd for $C_{30}H_{31}N_2O_3^+$ 467.2329 m/z, found, 467.2346 m/z.

Synthesis of hNR1

hNR (567 mg, 1 mmol) and HBTU (379 mg, 1 mmol) were dissolved in DMF (10 mL), followed by the addition of DIPEA (495 μL, 3 mmol). The mixture was stirred for 0.5 hours. Subsequently, tert-butyl sarcosinate hydrochloride (272 mg, 1.5 mmol) was added, and the reaction mixture was stirred continuously for 24 hours. After the reaction was complete, the mixture was poured into deionized H_2O (50 mL), then the precipitate was filtered and washed with H_2O. The precipitate was dried and further purified by silica gel column chromatograph (DCM/MeOH, 200/1) to afford hNR1 as a dark purple solid (501 mg, 72 % yield). HRMS (ESI$^+$): calcd for $C_{37}H_{44}N_3O_4^+$ 594.3326 m/z, found, 594.3337 m/z.

Synthesis of hNR2

hNR1 (694 mg, 1 mmol) was dissolved in anhydrous DCM (10 mL), followed by the addition of TFA (1 mL). The solution was stirred at room temperature for 3 hours. The solvent was removed under vacuum and the residue was purified by silica gel column chromatography (DCM/MeOH, 180/1) to afford hNR2 as a dark purple solid (554 mg, 87 %). HRMS (ESI$^+$): calcd for $C_{33}H_{36}N_3O_4^+$ 538.2700 m/z, found, 538.2731 m/z.

Synthesis of hNR3

hNR2 (319 mg, 0.5 mmol) and HBTU (190 mg, 0.5 mmol) were dissolved in DMF (10 mL), followed by the addition of DIPEA (1.5 mmol). The mixture was stirred for 0.5 hours. Subsequently, N-Boc-ethylenediamine (136 mg, 0.75 mmol) was added, and the reaction mixture was stirred continuously for 24 hours. After the reaction was complete, the mixture was poured into deionized H_2O (50 mL), and the precipitate was filtered and washed with H_2O. The dried precipitate was further purified through silica gel chromatograph column (DCM/MeOH, 200/1) to afford hNR3 as a dark purple solid (294 mg, 75 %). HRMS (ESI$^+$): calcd for $C_{40}H_{50}N_5O_5^+$ 680.3806 m/z, found, 680.3816 m/z.

Synthesis of hNR-NH_2

hNR3 (156 mg, 0.2 mmol) was dissolved in anhydrous DCM (5 mL), followed by the addition of TFA (0.5 mL). The solution was stirred at room temperature for 3 hours. The solvent was removed under vacuum, and the residue was

purified by silica gel column chromatography (DCM/MeOH, 100/1) to afford hNR-NH_2 as a dark purple solid (102 mg, 75 %). HRMS (ESI$^+$): calcd for $C_{35}H_{42}N_5O_3^+$ 580.3282 m/z, found, 580.3309 m/z.

(1)

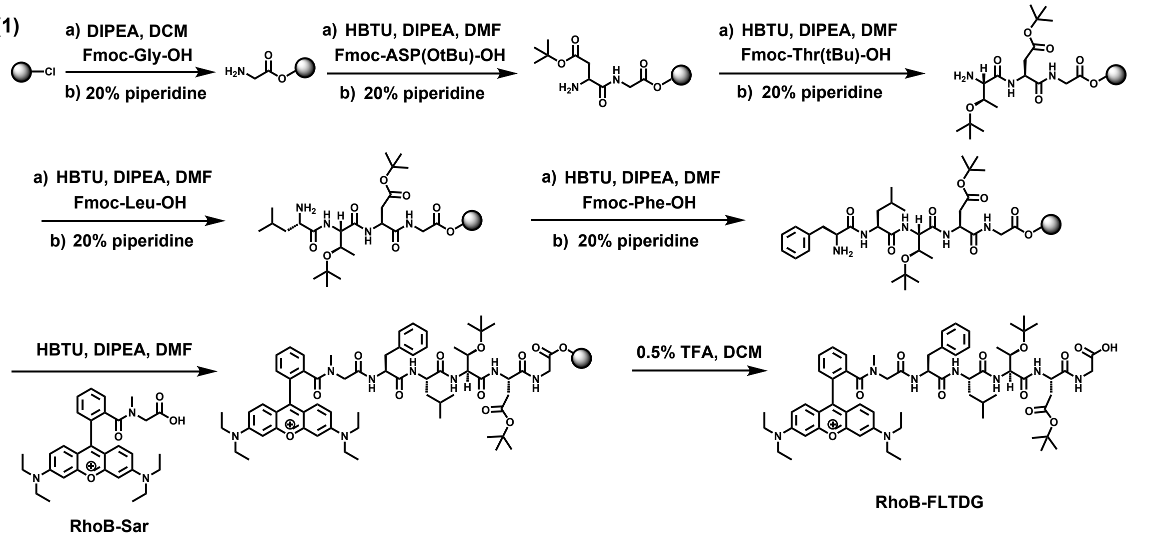

(2)

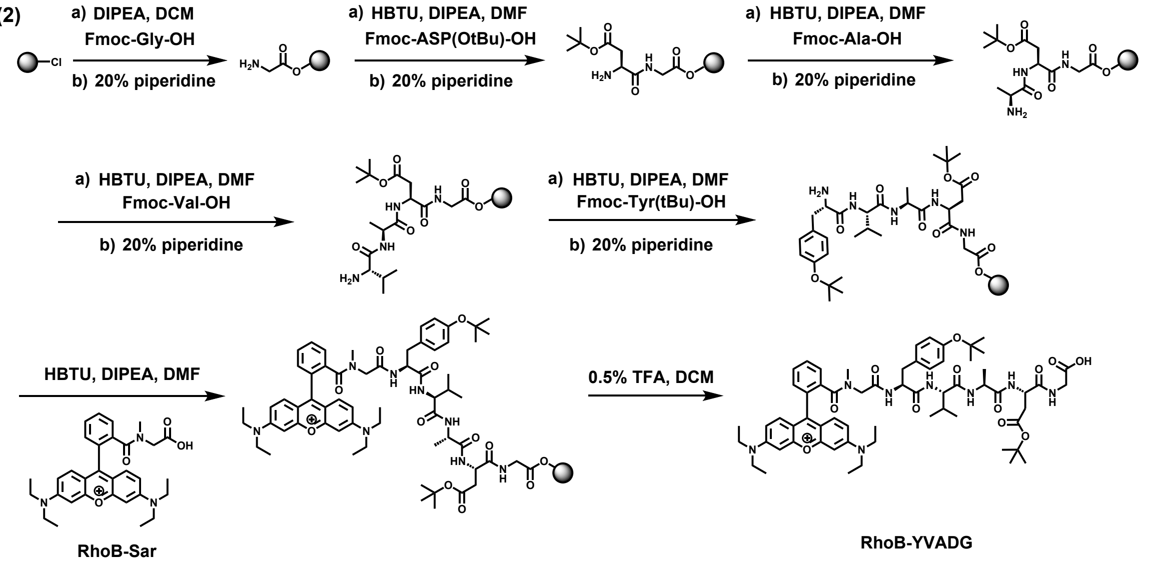

(3)

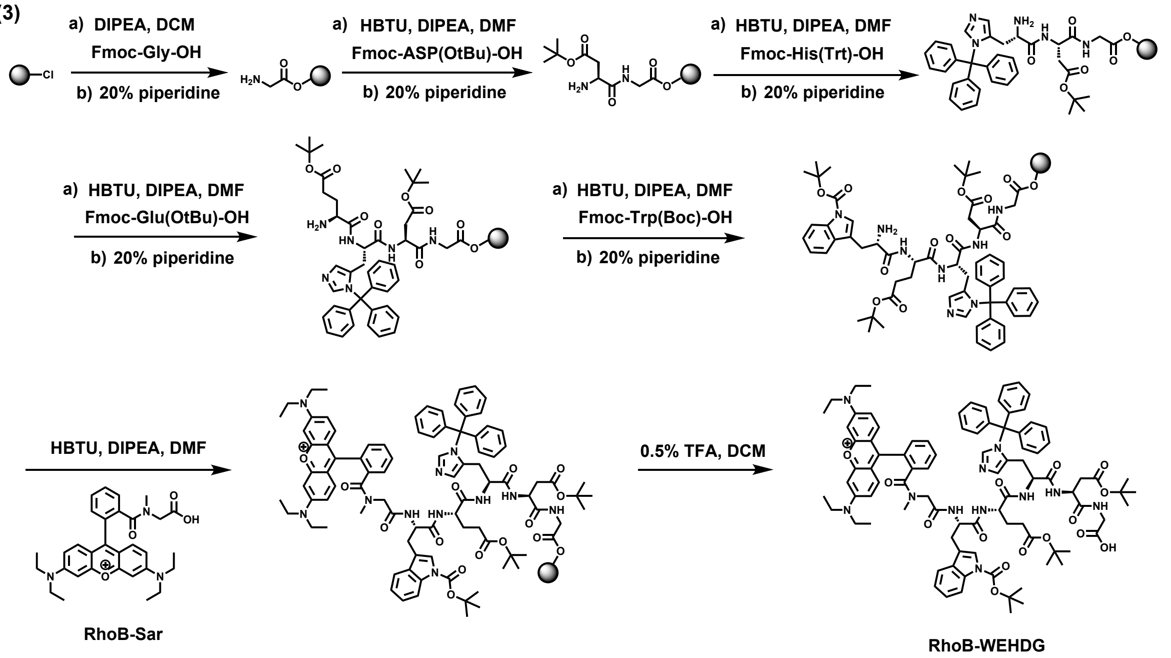

Scheme S2. Solid phase synthesis

Synthesis of RhoB-FLTDG

The peptide RhoB-Phe-Leu-Thr(tBu)-Asp(OtBu)-Gly-COOH (RhoB-FLTDG) was synthesized *via* a solid phase synthesis approach (SPPS). Briefly, the first amino acid was loaded onto the 2-chlorotrityl chloride resin. Subsequently, the capping reagent (DCM/MeOH/DIPEA = 17/2/1, 20 mL) was applied to react with unreacted active sites on the resin. The Fmoc group was then removed using DMF solution containing 20 % piperidine. The next Fmoc-protected amino acid was coupled to the free amino group using HBTU. Through the SPPS approach, 0.4 mmol Fmoc-Gly-OH (119 mg), Fmoc-Asp(OtBu)-OH (164 mg), Fmoc-Thr(tBu)-OH (159 mg), Fmoc-Leu-OH (141 mg), Fmoc-Phe-OH (154 mg) and RhoB-Sar (220 mg) were sequentially added to 2-chlorotrityl chloride (0.5 g) to form RhoB-FLTDG. The peptide was cleaved from the resin using 0.5 % TFA/DCM, and the collected eluent was concentrated under reduced pressure. Finally, cold diethyl ether (50 mL) was added, and the resulting precipitate was filtered to obtain a pink solid. The crude product RhoB-FLTDG was directly used in the next step without further purification (305 mg, 64 % yield). HRMS (ESI$^+$): calcd for $C_{64}H_{87}N_8O_{12}^+$ 1159.6438 m/z, found, 1159.6442 m/z.

Synthesis of RhoB-YVADG

Using an SPPS approach, 0.4 mmol Fmoc-Gly-OH (119 mg), Fmoc-Asp(OtBu)-OH (164 mg), Fmoc-Ala-OH (124 mg), Fmoc-Val-OH (136 mg), Fmoc-Tyr(tBu)-OH (184 mg) and RhoB-Sar (220 mg) were sequentially added to 2-chlorotrityl chloride (0.5 g) to form RhoB-YVADG. The peptide was cleaved from the resin using 0.5% TFA/DCM, and the collected eluent was concentrated under reduced pressure. Cold diethyl ether (50 mL) was then added, and the resulting precipitate was filtered to obtain a pink solid (316 mg, 68 % yield). The crude product RhoB-YVADG was directly used in the next step without further purification. HRMS (ESI$^+$): calcd for $C_{62}H_{83}N_8O_{12}^+$ 1131.6125 m/z, found, 1131.6122 m/z.

Synthesis of RhoB-WEHDG

Using an SPPS approach, 0.4 mmol Fmoc-Gly-OH (119 mg), Fmoc-Asp(OtBu)-OH (164 mg), Fmoc-His(Trt)-OH (248 mg), Fmoc-Glu(OtBu)-OH (170 mg), Fmoc-Trp(Boc)-OH (210 mg) and RhoB-Sar (220 mg) were sequentially added to 2-chlorotrityl chloride (0.5 g) to form RhoB-WEHDG. The peptide was cleaved from resin using 0.5% TFA/DCM, and the collected eluent was concentrated under reduced pressure. Cold diethyl ether (50 mL) was then added, and the resulting precipitate was filtered to obtain a pink solid (358 mg, 55 % yield). The crude product RhoB-WEHDG was directly used in the next step without further purification. HRMS (ESI$^+$): calcd for $C_{91}H_{106}N_{11}O_{15}^+$ 1593.7896 m/z, found, 1593.7901 m/z.

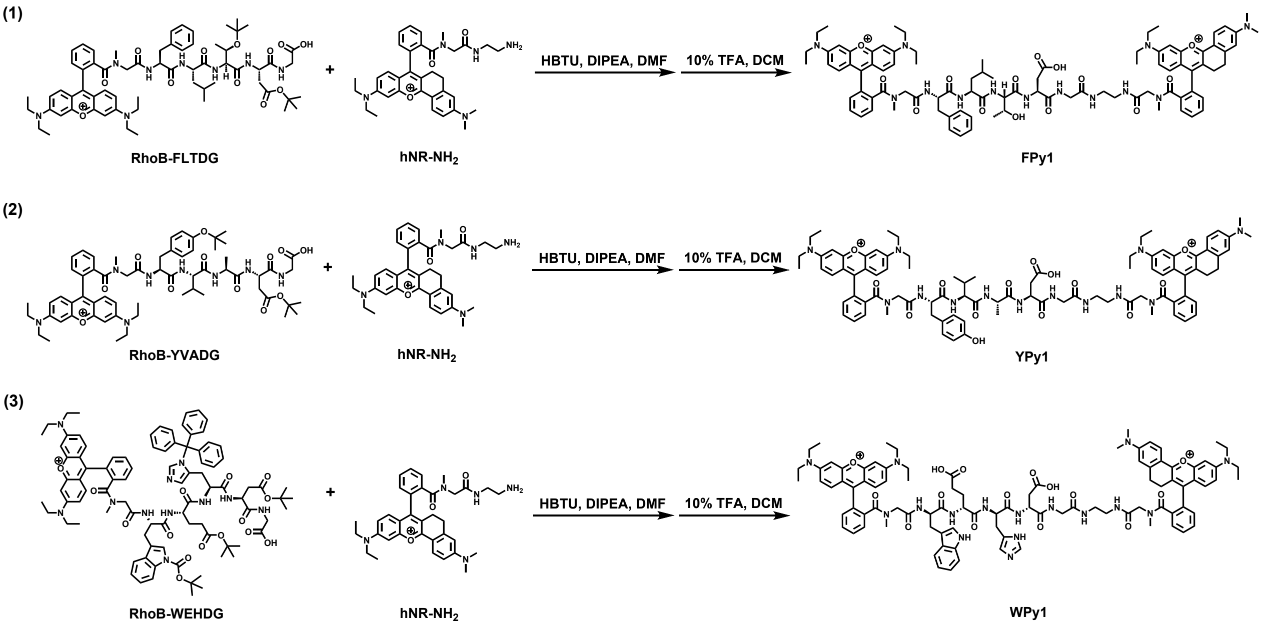

Scheme S3. Acceptor conjugation and side chain deprotection to make the fluorescent probes.

Synthesis of FPy1

RhoB-FLTDG (232 mg, 0.2 mmol) and HBTU (76 mg, 0.2 mmol) were dissolved in DMF (10 mL), followed by the addition of DIPEA (99 µL, 0.6 mmol). The mixture was stirred for 0.5 hours. Subsequently, hNR-NH_2 (272 mg, 0.4 mmol) was added, and the reaction mixture was stirred continuously for 24 hours. After the reaction was complete, the mixture was poured into deionized H_2O (50 mL), the precipitate was filtered and washed with H_2O. The dried precipitate was directly dissolved in anhydrous DCM (5 mL) without further purification, followed by the addition of TFA (0.5 mL). The reaction mixture was stirred for 3 hours. DCM was removed under vacuum, and the residue was purified by silica gel column chromatography (DCM/MeOH, 180/1) to afford **FPy1** as a dark purple solid (48 mg, 14 % yield). HRMS (ESI$^+$): calcd for $C_{91}H_{111}N_{13}O_{142}^{2+}$ 805.4197 m/z; found, 805.4203 m/z.

Synthesis of YPy1

RhoB-YVADG (226 mg, 0.2 mmol) and HBTU (76 mg, 0.2 mmol) were dissolved in DMF (10 mL), followed by the addition of DIPEA (99 µL, 0.6 mmol). The mixture was stirred for 0.5 hours. Subsequently, hNR-NH_2 (272 mg, 0.4 mmol) was added, and the reaction mixture and stirred continuously for 24 hours. After the reaction was complete, the mixture was poured into deionized H_2O (50 mL), and the precipitate was filtered and washed with H_2O. The dried precipitate was directly dissolved in anhydrous DCM (5 mL) without further purification, followed by the addition of TFA (0.5 mL). The reaction mixture was stirred for 3 hours. DCM was removed under vacuum and the residue was purified by silica gel column chromatography (DCM/MeOH, 180/1) to afford **YPy1** as a dark purple solid (33 mg, 10 % yield). HRMS (ESI$^+$): calcd for $C_{89}H_{107}N_{13}O_{142}^{2+}$ 791.4040 m/z; found, 790.9030 m/z.

Synthesis of WPy1

RhoB-WEHDG (319 mg, 0.2 mmol) and HBTU (76 mg, 0.2 mmol) were dissolved in DMF (10 mL), followed by the addition of DIPEA (99 µL, 0.6 mmol). The mixture was stirred for 0.5 h. Subsequently, hNR-NH_2 (272 mg, 0.4 mmol) was added, and the reaction mixture was stirred continuously for 24 h. After the reaction was complete, the mixture was poured into deionized H_2O (50 mL), the precipitate was filtered and washed with H_2O. The dried precipitate was directly dissolved in anhydrous DCM (5 mL) without further purification, followed by the addition of TFA (0.5 mL). The reaction

mixture was stirred for 3 h. DCM was removed under vacuum, and the residue was purified by silica gel column chromatography (DCM/MeOH, 180/1) to afford **WPy1** as a dark purple solid (21 mg, 6 % yield). HRMS (ESI^+): calcd for $C_{94}H_{108}N_{16}O_{152}^{2+}$ 850.9100 m/z; found, 850.9097 m/z.

Analysis of probe purity by LC-MSD

Prepare dilute solutions of **FPy1**, **YPy1** and **WPy1** using chromatographic-grade acetonitrile and transfer them to sample vials. Begin with an initial mobile phase consisting of 20% acetonitrile (0.05 % formic acid) and 80% H_2O (0.05 % formic acid). Over the course of 30 minutes, implement a gradient change to a mobile phase of 90% acetonitrile (0.05 % formic acid) and 10% H_2O (0.05 % FA). Under the elution conditions, LC-MSD was performed on the above probe solutions and blank solvent acetonitrile.

Sensitivity of probes for caspase-1

To investigate the sensitivity of the probes for caspase-1, varying concentration of caspase-1 (0, 0.2, 0.4, 0.6, 0.8, 1.0, 1.25, 1.5, 1.75, 2, 2.5, 3 U/mL) were incubated with **FPy1** (5 μM), **YPy1** (5 μM) and **WPy1** (5 μM) respectively in caspase-1 assay buffer (50 mM HEPES, 50 mM NaCl, 0.1 % chaps, 5 % glycerol, 10 mM DTT, 10 mM EDTA) at 37 °C for 2 hours. Fluorescence spectra were recorded using Hitachi F-2700 spectrophotometer with an excitation at 510 nm (Photomultiplier voltage 700 V; Slit widths ex: 10 nm and em =5.0 nm). The fluorescence intensity of **FPy1**, **YPy1** and **WPy1** at 585 nm were plotted against the concentration of caspase-1, and a linear regression fitted from the corresponding concentration of caspase-1, and linear regression was applied to determine the slope k. The limit of detection (LoD) was calculated from 3σ/k, in which σ represents the standard deviation of 15 blank measurements.

Enzyme kinetic studies

Various concentrations of **FPy1**, **YPy1** and **WPy1** (0.5, 1, 2, 4 μM) in 100 μL enzyme assay buffer (50 mM HEPES, 50 mM NaCl, 0.1 % chaps, 5 % glycerol, 10 mM DTT, 10 mM EDTA) were placed in a 96-well black plate. The reaction in each well was initiated by the addition of recombinant human caspase-1 (2 U/mL in 100 μL enzyme assay buffer). The fluorescence intensity at 585 nm was measured on a Tecan Infinite M1000 Multi-function microplate reader (λ_{ex} = 510 nm) at 37 °C every 2 minutes. The K_M value of probes was determined from the Lineweaver-Burk plot.

Reaction time of probes for caspase-1

To assess the reaction time of the probes for caspase-1, the fluorescence spectra of **FPy1**, **YPy1** and **WPy1** incubated with caspase-1 in assay buffer at 37 °C were recorded at time intervals of 0, 5, 10, 20, 30, 40, 50, 60, 80 and 100 minutes using a Hitachi F-2700 spectrophotometer with an excitation at 510 nm (Photomultiplier voltage 700 V; Slit widths ex: 10 nm and em =5.0 nm). The fluorescence intensity of **FPy1**, **YPy1** and **WPy1** at 585 nm were plotted separately against the reaction time.

Selectivity of probes for caspase-1

The selectivity of the probes for caspase-1 was evaluated by incubating **FPy1**, **YPy1** and **WPy1** with various biomolecules, including HSA (100 μg/mL), BSA (100 μg/mL), GSH (1 mM), Glucosidase (100 μg/mL), Lipase (100 μg/mL), Collagenase (100 μg/mL), Thrombin (100 μg/mL), ATP (1 mM), ADP (1 mM), H_2O_2 (1 mM), ClO^-(1 mM), Caspase-3 (1 μg/mL), Caspase-4 (2 U/mL), Caspase-8 (2 U/mL)and Caspase-1 (2 U/mL) in assay buffer. The mixture was incubated at 37 °C for 2 hours and fluorescence spectra were recorded using a Hitachi F-2700 spectrophotometer with excitation at 510 nm, for each group respectively (Photomultiplier voltage 700 V; Slit widths ex: 10 nm and em =5.0 nm). The fluorescence intensity of **FPy1**, **YPy1** and **WPy1** at 585 nm was plotted against the reaction time.

Cytotoxicity assay (CCK-8)

The cytotoxicity of **FPy1** and **YPy1** to THP-1 was evaluated by cell counting kit-8 (CCK-8). Briefly, the THP-1 cells were seeded in 96-well plates (5000 cells/well) and cultured at 37 °C for 24 hours. Various concentrations of **FPy1** or **YPy1** (0, 2.5, 5, 10, 20, 30, 40, 50 μM) in RPMI 1640 medium were added, and the cells were kept incubation for 12 hours. Then, the cells were washed with PBS and incubated in the 100 μL fresh medium with 10 μL CCK8 dye for an additional 2 hours. The absorbance was measured at 450 nm using a Tecan Infinite M1000 Multi-function microplate reader, and cell viability was calculated using the formula:

$$\text{Cell viability (\%)} = [(OD_{test}-OD_{blank})/(OD_{control}-OD_{blank})] \times 100\%$$

Intracellular imaging with FPy1 in THP-1 cells

THP-1 cells were seeded in dishes and cultured overnight. After stimulation with LPS (1 μg/mL in RPMI 1640 medium) for 4 hours, the cells were further treated with Nig (10 μM) or ATP (2 mM) for 0, 1, 2 hours. The medium was then replaced with fresh medium with **FPy1** (5 μM), and cells were incubated at 37 °C for 1 hour before being washed twice with PBS. The fluorescence imaging was performed under the excitation at 514 nm with signal collection in the range of 530-610 nm.

Intracellular imaging with YPy1 in THP-1 cells

THP-1 cells were seeded in dishes and cultured overnight. After stimulation with LPS (1 μg/mL in RPMI 1640 medium) for 4 hours, the cells were further treated with Nig (10 μM) or ATP (2 mM) for 0, 1, 2 hours. The medium was then replaced with fresh medium with **YPy1** (5 μM), and cells were incubated at 37 °C for 1 hour before being washed twice with PBS. The fluorescence imaging was performed under the excitation at 514 nm with signal collection in the range of 530-610 nm.

Subcellular Localization of FPy1

THP-1 cells were seeded in dishes and cultured overnight. After stimulation with LPS (1 μg/mL in RPMI 1640 medium) for 4 hours, the cells were further treated with Nig (10 μM) for 2 hours. The medium was then replaced with fresh medium with **FPy1** (5 μM), and cells were incubated at 37 °C for 1 hour. After washing, the THP-1 cells were separately incubated with commercially available dyes targeting mitochondria (Mito-Tracker Green), lysosomes (Lyso-Tracker Green), endoplasmic reticulum (ER-Tracker Green), and Golgi apparatus (Golgi-Tracker Green) for 30 minutes. The distribution of **FPy1** was analyzed by collecting organelle-specific dye fluorescence at 495-525 nm with 488 nm excitation, while simultaneously detecting **FPy1** fluorescence at 530-610 nm with 514 nm excitation.

Nucleus pulposus spheroid cultivation

Nucleus pulposus cells were isolated from discs and resuspended in 1mL DMEM/F12 medium containing 10 μM ITS-A. A total of 10^5 cells were counted and transferred into centrifuge tubes. The cells were incubated in these tubes with half of the medium changed periodically. Cell spheroids were collected once nano-sized cell were observed in the tubes.

Intracellular imaging in nucleus pulposus cells and 3D cell spheroids

The primary nucleus pulposus cells or 3D cell spheroids were seeded in dishes and cultured overnight. Subsequently, the cells or spheroids were incubated with H_2O_2 for 30, 60 and 90 minutes. Afterward, the cells were further incubated in medium containing 5 μM **FPy1** for 1 hour. Fluorescence imaging was then performed with an excitation of 514 nm,

and signals were collected from 530 to 610 nm.

Chondrocytes spheroid cultivation

Chondrocytes were isolated from articular cartilage and resuspended in 1mL DMEM/F12 medium containing 10 μM ITS-A. A total of 10^5 cells were counted and transferred into centrifuge tubes. The cells were incubated in these tubes with half of the medium changed periodically. Cell spheroids were collected once nano-sized cell were observed in the tubes.

Intracellular imaging in chondrocytes and 3D cell spheroids

The chondrocytes or 3D cell spheroids were seeded in dishes and cultured overnight. Subsequently, the cells or spheroids were incubated with H_2O_2 for 30, 60 and 90 minutes. Afterward, the cells were further incubated in medium containing 5 μM **FPy1** for 1 hour. Fluorescence imaging was then performed with an excitation of 514 nm, and signals were collected from 530 to 610 nm.

Flow cytometry assays in THP-1 cells

The flow cytometry assays were performed to evaluate the response of **FPy1** in THP-1 cells. THP-1 cells were seeded in 6-well plates at a density of 10^5 cells per well and cultured overnight. The treatment protocol for each group followed the same steps as those used for fluorescent imaging. After treatment, the cells were gently rinsed three times with PBS, resuspended in PBS, and then added to flow cytometry tubes placed on ice. The results were analyzed with using FlowJo software.

Western blotting

Cells were rinsed with cold PBS, lysed in 5× sodium dodecyl sulfate (SDS) gel loading buffer and boiled for 10 minutes. Proteins in the lysate were then separated on 12% SDS polyacrylamide gels, electro-transferred to polyvinylidene difluoride (PVDF) membranes (Millipore, Boston), and stained with the following primary antibodies: cleaved caspase-1 (1:1000, Affinity, AF4005), β-actin (1:1000, Abcam, ab8226). Primary antibodies were stained with horseradish peroxidase-conjugated secondary antibodies (1:10000, Abcam, ab205718) and visualized with a chemiluminescence ECL Western-blotting system (Millipore).

Immunofluorescence staining

Fixed cells or tissue sections were washed with PBS, permeabilized with 0.2 % Triton X-100 (Sigma–Aldrich) for 20 minutes, and blocked with 5 % bovine serum albumin for 1 hour at 37 °C. Cells or sections were then incubated overnight at 4 °C with primary antibodies: GSDMD (1:1000, Abcam, ab219800) and cleaved Caspase-1 (1:1000, Affinity, AF4005). This was followed by incubation with secondary antibodies conjugated to Alexa Fluor®488 (1:200) for 1 hour at room temperature. Nuclei were counterstained with 4′,6-diamidino-2-phenylindole (DAPI, Beyotime, C1006) for 15 minutes. Staining results were examined under a fluorescence microscope (Olympus, IX83-FV3000) and quantified using ImageJ (Bethesda, MD, V1.8.0, NIH).

Needle puncture-induced disc degeneration in rats

A disc degeneration model was established in thirty female Sprague-Dawley rats (3 months old, approximately 150 g), obtained from the institutional Animal. The rats were anesthetized intraperitoneally with 0.8 % (w/v) pentobarbital sodium (10 μL/g body weight). Intervertebral discs between coccyx vertebra 5 and 6, 6 and 7, 7 and 8 (Co5/6, Co6/7,

Co7/8) were identified and marked on the skin. Discs degeneration was induced by percutaneously puncturing using the marked disc with 29 G needle to a depth of about 2 mm. The needle was then rotated 360° and maintained inside the disc for 1 minute before removal. Rats received intradiscal injection with **FPy1** at 0, 1, 2 and 4 week post-modeling, targeting the Co5/6, Co6/7 and Co 7/8. Animals were sacrificed and disc tissues were collected for analysis.

Histological evaluation of disc degeneration

Harvested rat discs were fixed in 4 % buffered paraformaldehyde for 24 hours, decalcified in 10 % ethylenediaminetetraacetic acid (EDTA, Solarbio, E8030) solution for 1 month, embedded in paraffin, and sectioned at a thickness of 5 μm. Safranin O staining and hematoxylin and eosin (HE) staining were performed to assess the degree of disc degeneration. Stained sections were evaluated by three independent raters, blinded to the research protocol, and measurements were averaged for statistical analysis. Histological scoring was based on cellularity, morphology, structure and integrity of the annulus fibrosus and nucleus pulposus regions, with a score of 0-3 indicating normal disc, 4-10 indicating moderate degeneration, and 11-16 as severe degeneration.

In vivo fluorescence imaging of intervertebral disc degeneration

Twelve rats were modeled with needle puncture-induced intervertebral disc degeneration at the Co5/6, Co6/7 and Co 7/8 levels. Rats were intradiscally injected with **FPy1** at 0, 1, 2 and 4 weeks after modeling. In vivo fluorescence images were acquired using an in vivo Imaging System (PerkinElmer). Following imaging, animals were sacrificed, and disc tissues were collected for further analysis.

In vivo fluorescence imaging of osteoarthritis

Eight-week-old male C57BL/6J mice (n = 6) were anesthetized with an intraperitoneal injection of pentobarbital sodium. Both hind limbs were shaved and disinfected. Both hind limbs were shaved and disinfected, and surgical procedures were performed sequentially on each knee joint. A medial parapatellar incision was made to expose the joint cavity, after which the anterior cruciate ligament was identified and completely transected using a microsurgical blade. The patella was then repositioned, and the joint capsule and skin were sutured. The same procedure was repeated on the contralateral knee. For the sham control group (n = 6), an identical surgical procedure was performed without ACL transection. All animals were allowed free movement in their cages after surgery. At 0, 7, 14, and 21 days post-surgery, the probe was injected into the joint cavity through an insulin syringe for in vivo fluorescence imaging.

High-content screening method

Mouse peritoneal macrophages were seeded in 96-well black plates at a density of 10^6 cells/well and cultured overnight. After stimulation with LPS for 4 hours, the cells were further incubated in medium containing Nig (10 μM) and various natural compounds (10 μM) for 2 hours. Subsequently, the medium was removed, and cells were stained with 5 μM **FPy1**. Fluorescent imaging and quantitative analysis were performed using a high-content screening system, with an excitation filter of 514 nm and a collection wavelength range of 540-600 nm.

Supplementary Figures

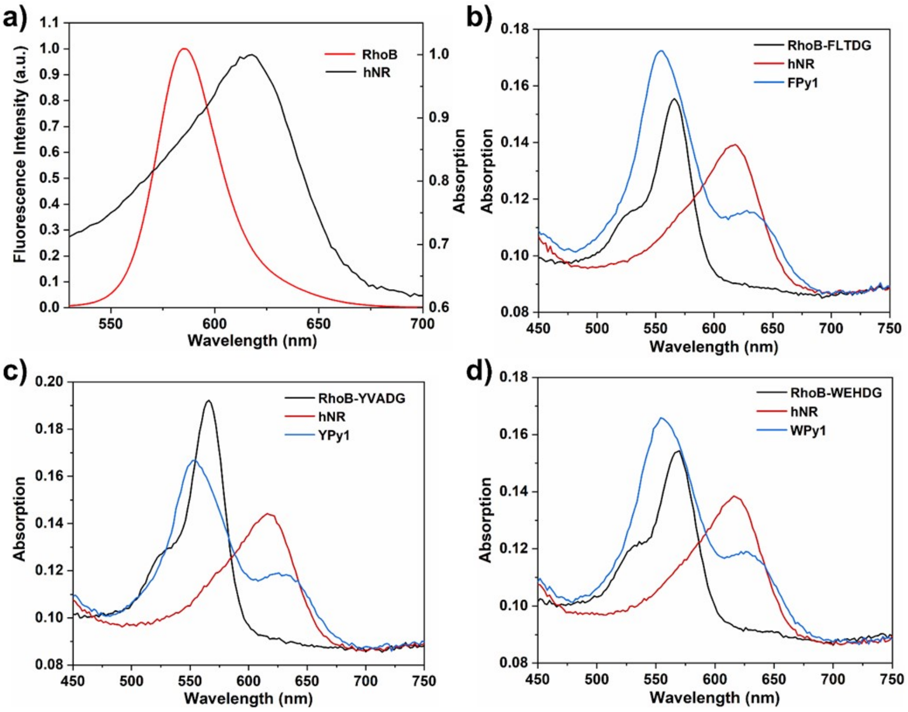

Figure S1. (a) Normalized absorption spectra of hNR and fluorescence emission spectra of RhoB. (b) Absorption spectra of RhoB-FLTDG (5 μM), hNR (5 μM) and **FPy1** (5 μM) in aqueous solution (H_2O:DMSO = 99:1, v/v). (c) Absorption spectra of RhoB-YVADG (5 μM), hNR (5 μM) and **YPy1** (5 μM) in aqueous solution (H_2O:DMSO = 99:1, v/v). (d) Absorption spectra of RhoB-WEHDG (5 μM), hNR (5 μM) and **WPy1** (5 μM) in aqueous solution (H_2O:DMSO = 99:1, v/v).

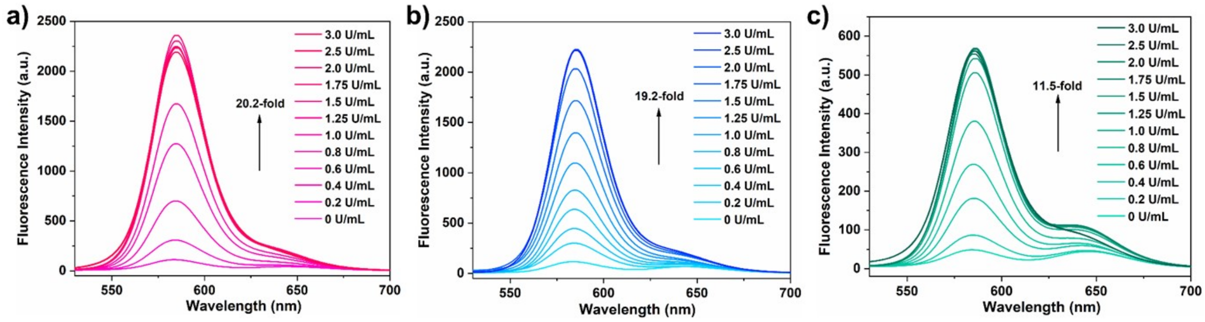

Figure S2. Fluorescence spectra of (a) **FPy1** (5 μM), (b) **YPy1** (5 μM) and (c) **WPy1** (5 μM) after incubation with different concentration of caspase-1 in the assay buffer at 37 °C for 2 h. λ_{ex} = 510 nm; Photomultiplier voltage 700 V; excitation width = 10 nm, emission width = 5.0 nm.

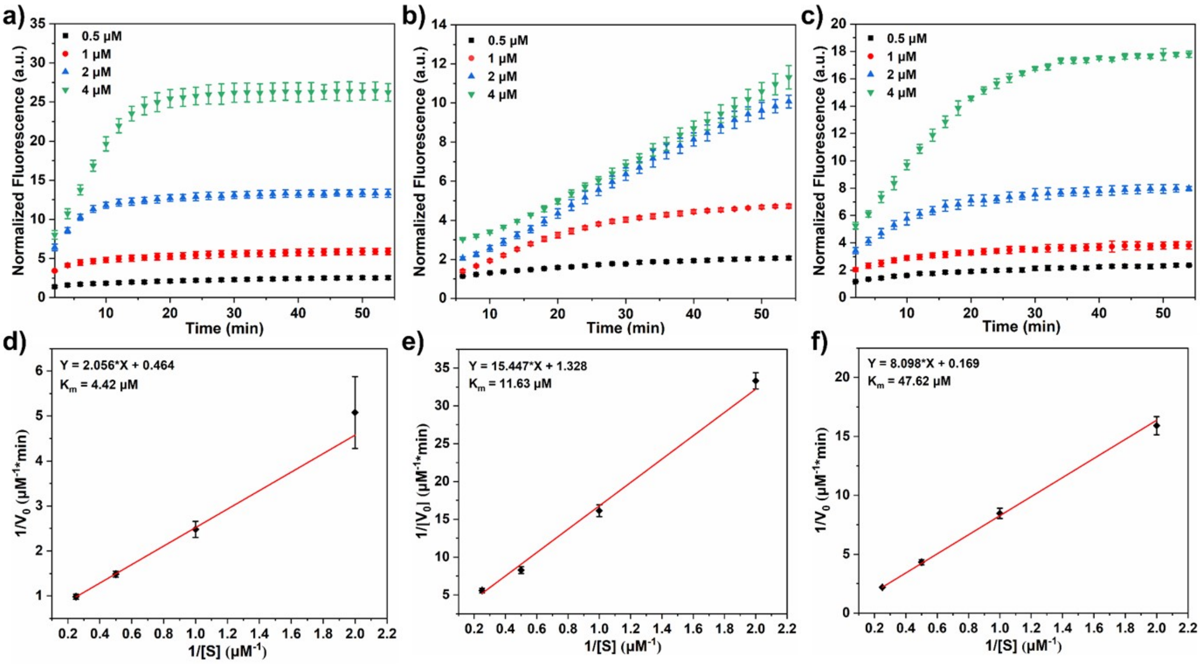

Figure S3. Kinetic evaluation of the caspase-1 enzyme-catalyzed reaction. (a) fluorescence intensity change of varying concentrations of **FPy1** (0.5-4 μM) incubated with caspase-1 (2U/mL) at 37 °C for indicated time. (b) fluorescence intensity change of varying concentrations of **YPy1** (0.5-4 μM) incubated with caspase-1 (2U/mL) at 37 °C for indicated time. (c) fluorescence intensity change of varying concentrations of **WPy1** (0.5-4 μM) incubated with caspase-1 (2U/mL) at 37 °C for indicated time. (d) Lineweaver-Burk plots of 1/[V_0] vs. 1/[S] for **FPy1**. (e) Lineweaver-Burk plots of 1/[V_0] vs. 1/[S] for **YPy1**. (f) Lineweaver-Burk plots of 1/[V_0] vs. 1/[S] for **WPy1**. λ_{ex} = 510 nm, λ_{em} = 585 nm

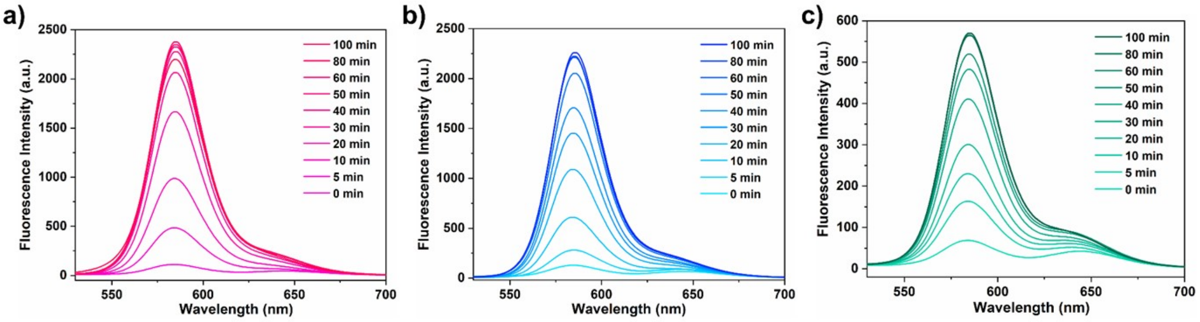

Figure S4. Time-dependent fluorescence spectra of 5 μM **FPy1** (a), **YPy1** (b) and **WPy1** (c) treated with caspase-1 in the assay buffer at 37 °C. λ_{ex} = 510 nm; Photomultiplier voltage 700 V; Slit widths ex: 10 nm and em =5.0 nm

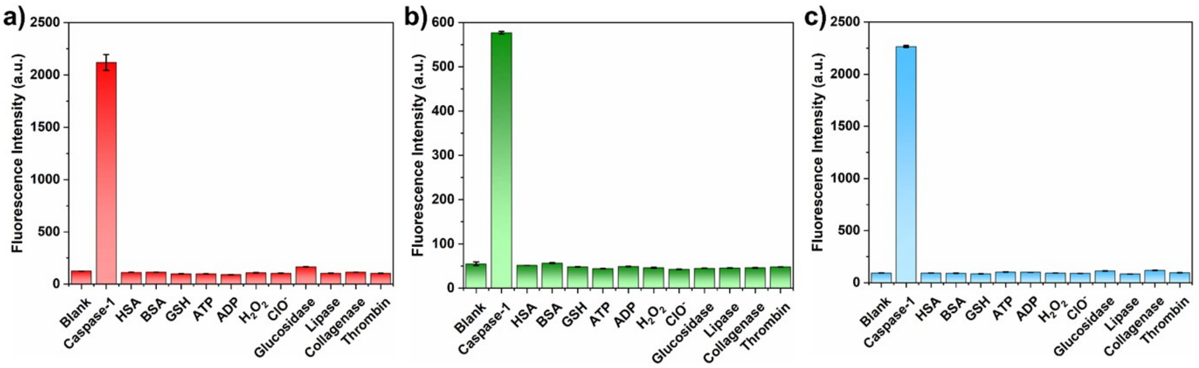

Figure S5. Fluorescence intensity of **FPy1** (a), **YPy1** (b) and **WPy1** (c) in response to biological interfering substances for 2h respectively, including caspase-1 (2 U/mL), HSA (100 μg/mL), BSA (100 μg/mL), GSH (1 mM), ATP (1 mM), ADP (1 mM), H_2O_2 (1 mM), ClO^- (1 mM), Glucosidase (100 μg/mL), Lipase (100 μg/mL), Collagenase (100 μg/mL), Thrombin (100 μg/mL). λ_{ex} = 510 nm, λ_{em} = 585 nm; Photomultiplier voltage 700 V; Slit widths ex: 10 nm and em = 5.0 nm

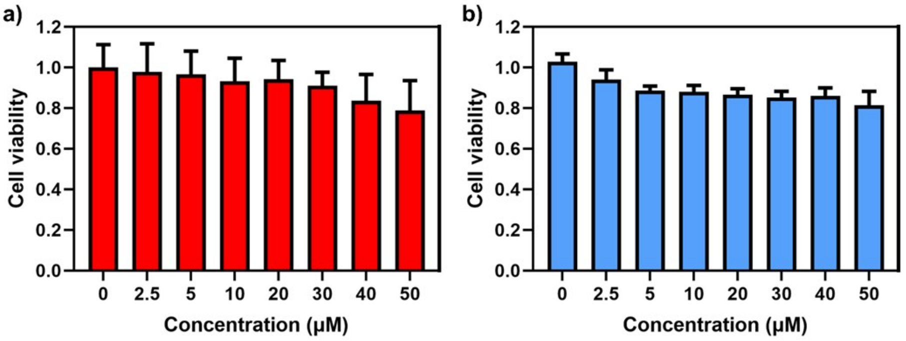

Figure S6. (a) The survival rates of THP-1 cells after incubation with different concentrations of **FPy1** (0-50 μM) for 12 h at 37 °C. (b) The survival rates of THP-1 cells after incubation with different concentrations of **YPy1** (0-50 μM) for 12 h at 37 °C.

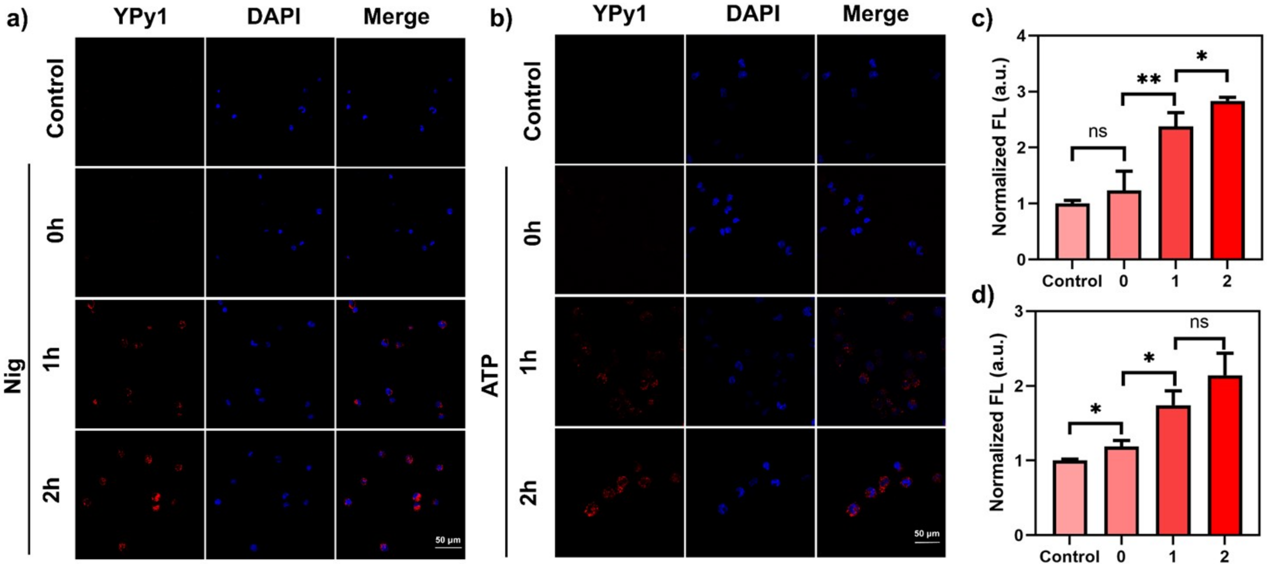

Figure S7. Bioimaging of Cas-1 in THP-1 cells stained with **YPy1**. (a) Confocal fluorescence images of THP-1 cells stained with **YPy1**. LPS serves as the first signal, priming the cells by upregulating NLRP3 and pro-caspase-1, while nigericin acts as the second signal to trigger NLRP3 inflammasome assembly and caspase-1 activation. First row, control group; second row, LPS-treated cells without nigericin stimulation; third row, LPS-treated cells stimulated with nigericin (10 μM) for 1h; fourth row, LPS-treated cells stimulated with nigericin (10 μM) for 2h. **YPy1** (5 μM, λ_{ex} = 514 nm, collected 530-610 nm); DAPI (1 μg/mL, λ_{ex} = 405 nm, collected 420-470 nm). (b) Confocal fluorescence images of THP-1 cells stained with **YPy1** (5 μM). LPS provides the priming signal to upregulate NLRP3 and pro-caspase-1, and ATP acts as the second signal to activate the NLRP3 inflammasome and caspase-1. First row, control group; second row, LPS-treated cells without ATP stimulation; third row, LPS-treated cells stimulated with ATP (2 mM) for 1h; fourth row, LPS-treated cells stimulated with ATP (2 mM) for 2h. **YPy1** (5 μM, λ_{ex} = 514 nm, collected 530-610 nm); DAPI (1 μg/ml, λ_{ex} = 405 nm, collected 420-470 nm). (c) Quantitative analysis of fluorescence intensity in (a). (d) Quantitative analysis of fluorescence intensity in (b). Statistical analyses were performed using one-way ANOVA with multiple comparisons. Significance levels are indicated as ns (not significant), *$P < 0.05$, **$P < 0.01$, ***$P < 0.001$, and ****$P < 0.0001$.

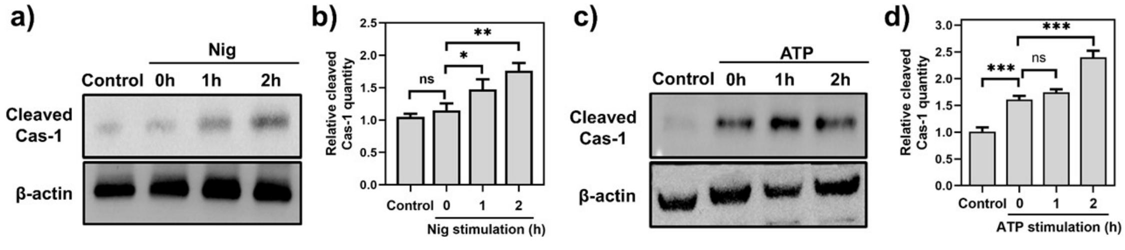

Figure S8. (a) Western blot analysis of cleaved caspase-1 levels in control and Nig-treated groups. (b) Relative grey levels of cleaved caspase-1 of THP-1 cells from panel S8a analyzed by ImageJ. (c) Western blot analysis of cleaved caspase-1 levels in control and ATP-treated groups. (d) Relative grey levels of cleaved caspase-1 of THP-1 cells from

panel S8c analyzed by ImageJ. Statistical analyses were performed using one-way ANOVA with multiple comparisons. Significance levels are indicated as ns (not significant), *P < 0.05, **P < 0.01, ***P < 0.001, and ****P < 0.0001.

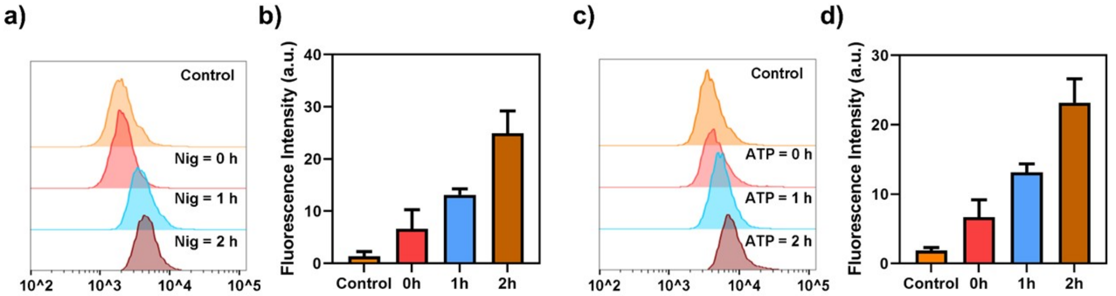

Figure S9. Flow cytometric analysis of **FPy1** fluorescence following NLRP3 inflammasome activation with nigericin (Nig) or ATP as secondary stimulants. (a) Flow cytometric analysis of **FPy1** fluorescence intensity in control and various model groups when Nig was used as the second stimulant in the classical NLRP3 inflammasome activation model. (b) Quantitative analysis of flow cytometric data for **FPy1** fluorescence in control and Nig-treated groups. (c) Flow cytometric profiles of **FPy1** fluorescence intensity in control and model groups with ATP as the second stimulant in the classical NLRP3 inflammasome activation model. (d) Quantitative analysis of flow cytometric data for **FPy1** fluorescence in control and ATP-treated groups.

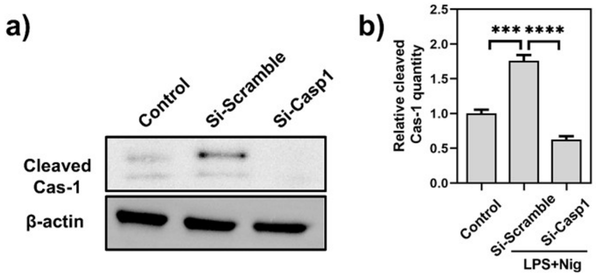

Figure S10. (a) Cleaved caspase-1 levels in Si-Scramble and Si-Casp1 groups that were examined by Western blots. (b) The relative ratio of the grey level of cleaved caspase-1 of THP-1 cells in panel S9a analyzed by ImageJ. Statistical analyses were performed with one-way ANOVA with multiple comparisons. Significance levels are indicated as ns (not significant), *P < 0.05, **P < 0.01, ***P < 0.001, and ****P < 0.0001.

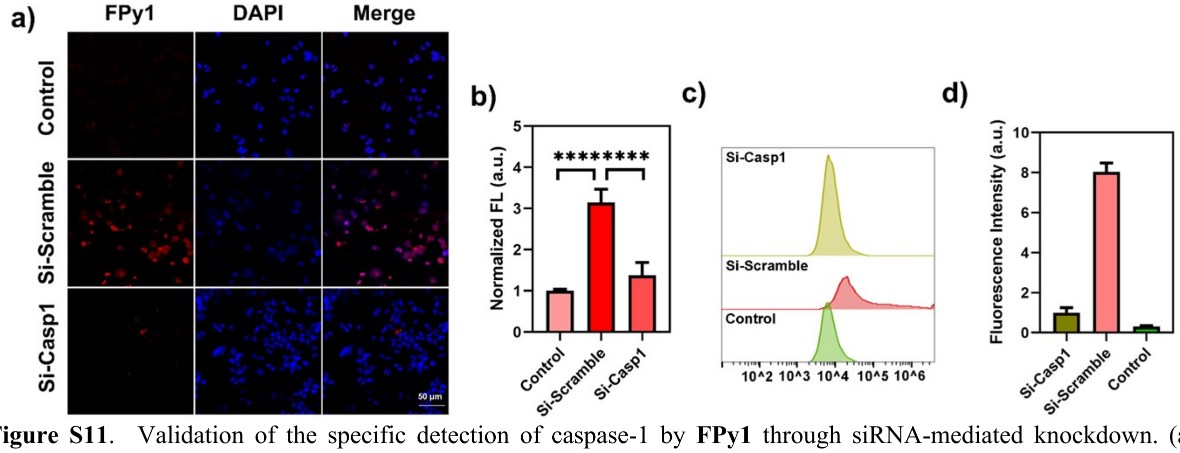

Figure S11. Validation of the specific detection of caspase-1 by **FPy1** through siRNA-mediated knockdown. (a) Confocal fluorescence images of THP-1 cells following knockdown treatment. Top row, control group; middle row, Nig-treated THP-1 cells transfected with non-caspase-1 (scramble) siRNA; bottom row, Nig-treated THP-1 cells transfected with caspase-1 siRNA. (b) Quantitative analysis of fluorescence intensity corresponding to the images in (a). (c) Flow cytometry analysis of **FPy1** fluorescence in siRNA-knockdown and non-knockdown groups. (d) Quantitative flow cytometric analysis of **FPy1** fluorescence in siRNA-knockdown and non-knockdown groups. Si-Casp1: THP-1 cells transfected with caspase-1 siRNA and sequentially stimulated with LPS (1 μg/mL) and Nig (10 μM); Si-Scramble: THP-1 cells transfected with non-targeting siRNA and sequentially stimulated with LPS (1 μg/mL) and Nig (10 μM); Control: untreated THP-1 cells. Fluorescence channels: DAPI (1 μg/ml, λ_{ex} = 405 nm, emission collected at 420-470 nm); **FPy1** (5 μM, λ_{ex} = 514 nm, emission collected at 530-610 nm). Statistical analysis was performed using one-way ANOVA with multiple comparisons. Significance levels are denoted as ns (not significant), *P < 0.05, **P < 0.01, ***P < 0.001, and ****P < 0.0001.

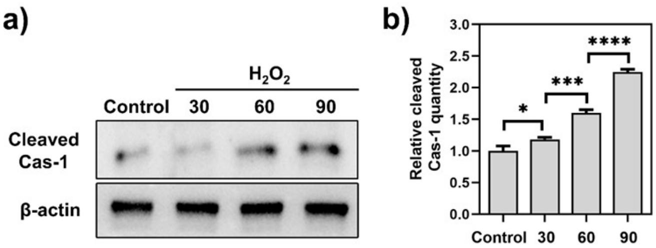

Figure S12. (a) Cleaved caspase-1 levels in control and H_2O_2-treated groups that were examined by Western blot. (b) The relative ratio of the grey level intensity of cleaved caspase-1 in nucleus pulposus cells in panel S10a, quantified using ImageJ. Statistical analysis was performed with one-way ANOVA with multiple comparisons. Significance levels are indicated as ns (not significant), *$P < 0.05$, **$P < 0.01$, ***$P < 0.001$, and ****$P < 0.0001$.

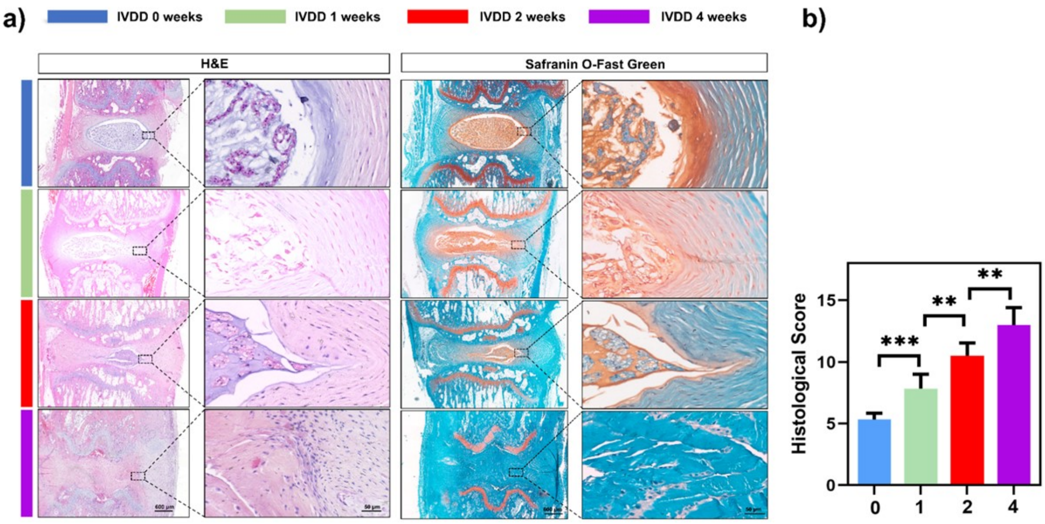

Figure S13. (a) Representative images of Hematoxylin-eosin staining and Safranine O/Fast Green staining in each group. (b) Quantification of histological score in the sections.

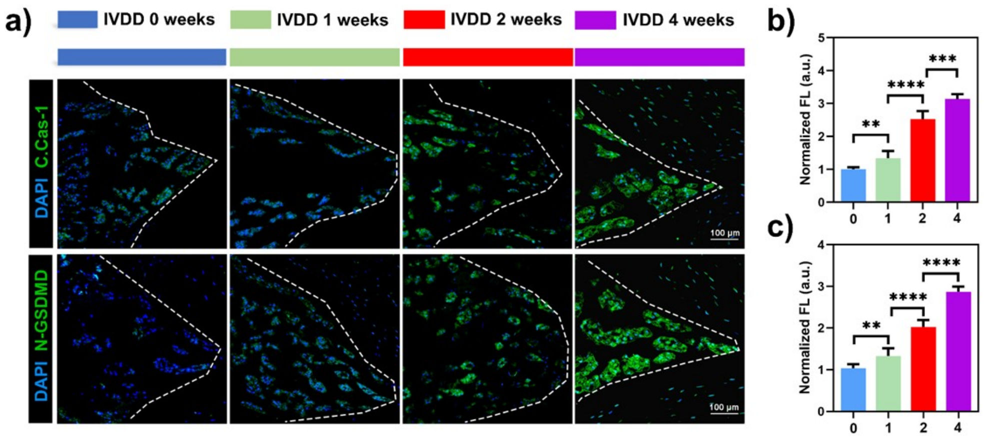

Figure S14. Immunofluorescence analysis of cleaved caspase-1 and N-GSDMD in intervertebral disc degeneration models at different stages. (a) Representative immunofluorescence images of cleaved caspase-1 and N-GSDMD of rat tails from each group. (b) Quantification analysis of immunofluorescence staining for cleaved caspase-1. (c) Quantification of immunofluorescence staining for N-GSDMD. Statistical analysis was performed using one-way ANOVA with multiple comparisons. Significance levels are denoted as ns (not significant), *$P < 0.05$, **$P < 0.01$, ***$P < 0.001$, and ****$P < 0.0001$. Green channel: λ_{ex} = 488 nm, emission collected at 500-550 nm; Blue channel: λ_{ex} = 405 nm, emission collected at 420-470 nm.

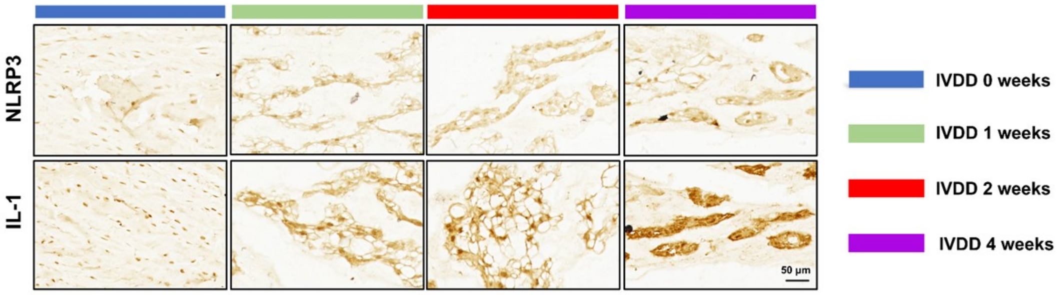

Figure S15. Representative immunohistochemistry images of NLRP3 inflammasome and IL-1 expression in rat caudal intervertebral discs at different stages of degeneration. Blue icons indicate the IVDD model with a 0 week; green icons indicate 1 week; red icons indicate 2 weeks; and purple indicate 4 weeks of degeneration.

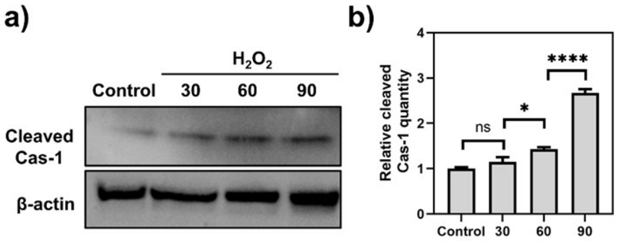

Figure S16. (a) Cleaved caspase-1 levels in control and H_2O_2-treated groups that were examined by Western blot. (b) The relative ratio of the grey level intensity of cleaved caspase-1 in primary chondrocytes in panel S10a, quantified using ImageJ. Statistical analysis was performed with one-way ANOVA with multiple comparisons. Significance levels are indicated as ns (not significant), *P < 0.05, **P < 0.01, ***P < 0.001, and ****P < 0.0001.

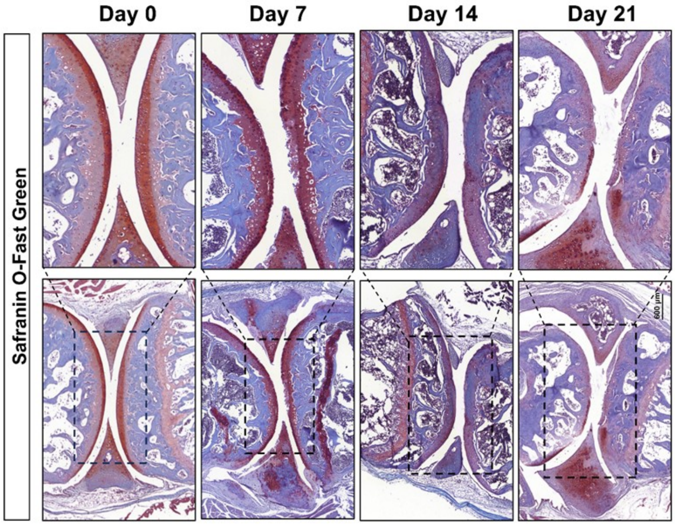

Figure S17. Representative images of Safranin O-fast green staining of osteoarthritis models at different stages. Top row: magnified views of Safranin O-fast green-stained sections; bottom row: overview of Safranin O-fast green-stained sections.

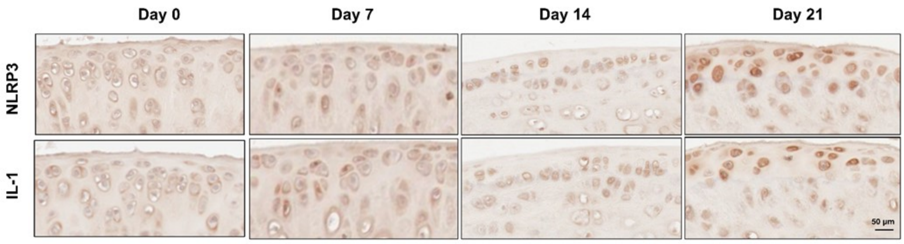

Figure S18. Representative immunohistochemistry images for NLRP3 inflammasome and IL-1 of knee joint within each group of the osteoarthritis model.

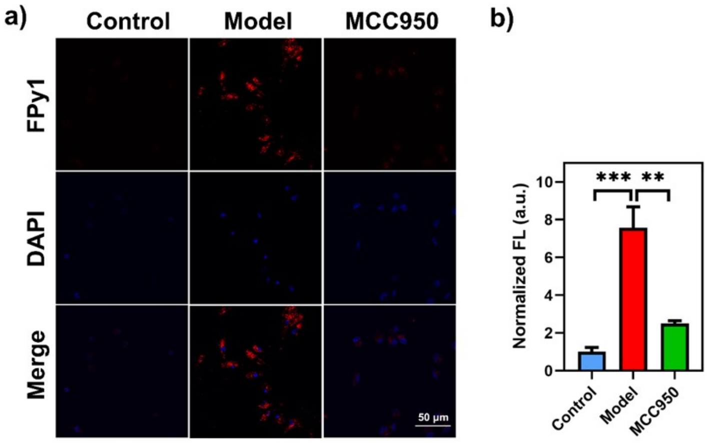

Figure S19. (a) Representative fluorescence images of primary peritoneal macrophages with different treatment. First column, control group; second column, LPS-treated cells were stimulated with Nig (10 μM) for 2h; third column, the positive drug group. (b) Quantitative analysis of **FPy1**' fluorescence intensity in (S15a). Statistical analysis was performed with one-way ANOVA with multiple comparisons. Significance levels are indicated as ns (not significant), *$P < 0.05$, **$P < 0.01$, ***$P < 0.001$, and ****$P < 0.0001$.

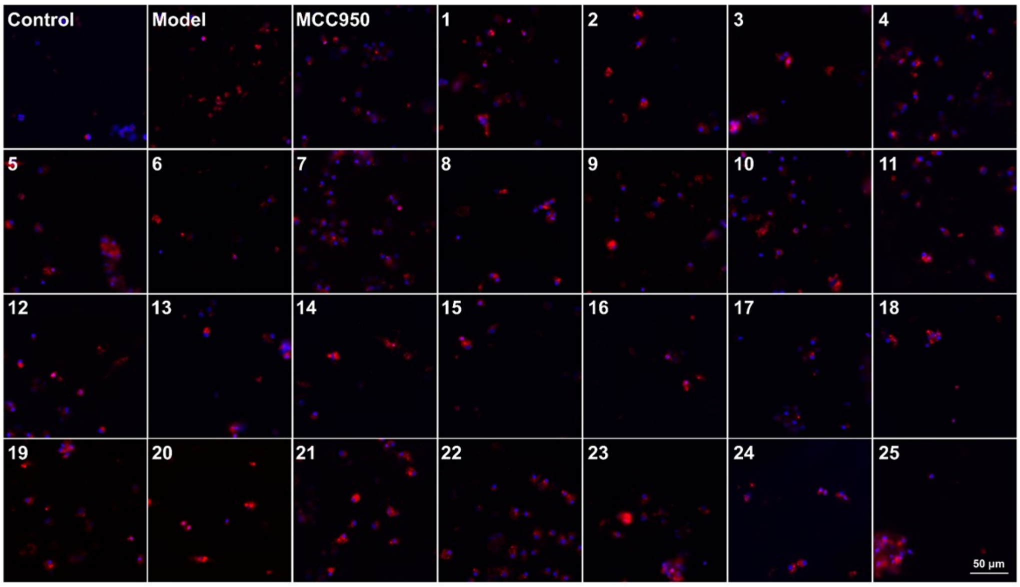

Figure S20. Representative fluorescence images of primary peritoneal macrophages under the intervention of natural products. The control group: cells were intact; the model group: cells were further stimulated with Nig (10 μM) after LPS (1μg/mL) stimulation; MCC950 group: LPS-treated cells were further co-treated with MCC950 (1 μM) for protection during Nig stimulation; 1 – 25 groups: LPS-treated cells were also further co-treated with 25 natural products (10 μM) during Nig stimulation. Blue channel: DAPI (λ_{ex} = 405 nm, emission collected at 420-470 nm); red channel: **FPy1** (λ_{ex} = 514 nm, emission collected at 530-610 nm).

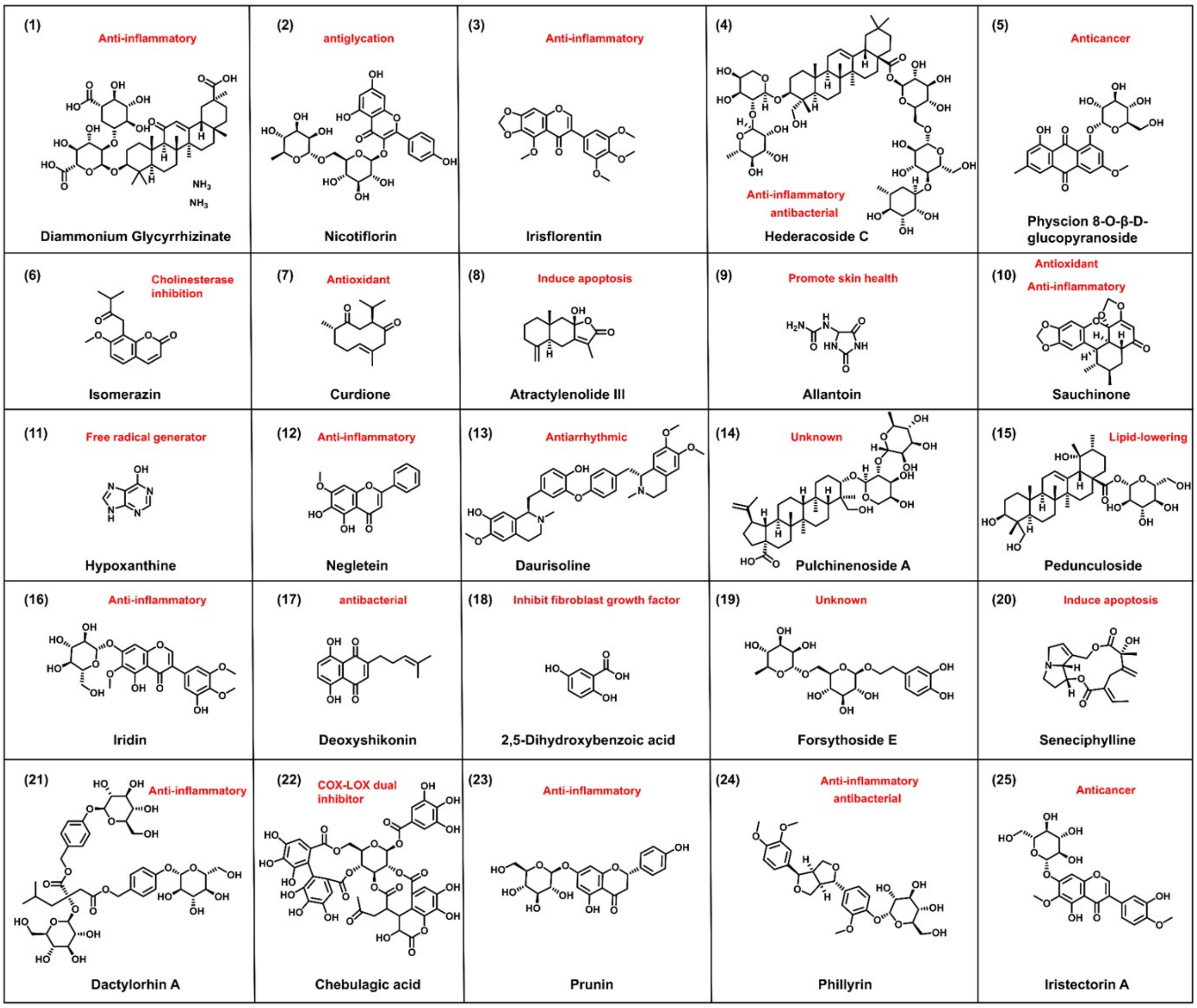

Figure S21. The names and structures of the 25 natural compounds used for the high-content screening. The red text indicates the reported biological activities of the corresponding compounds.

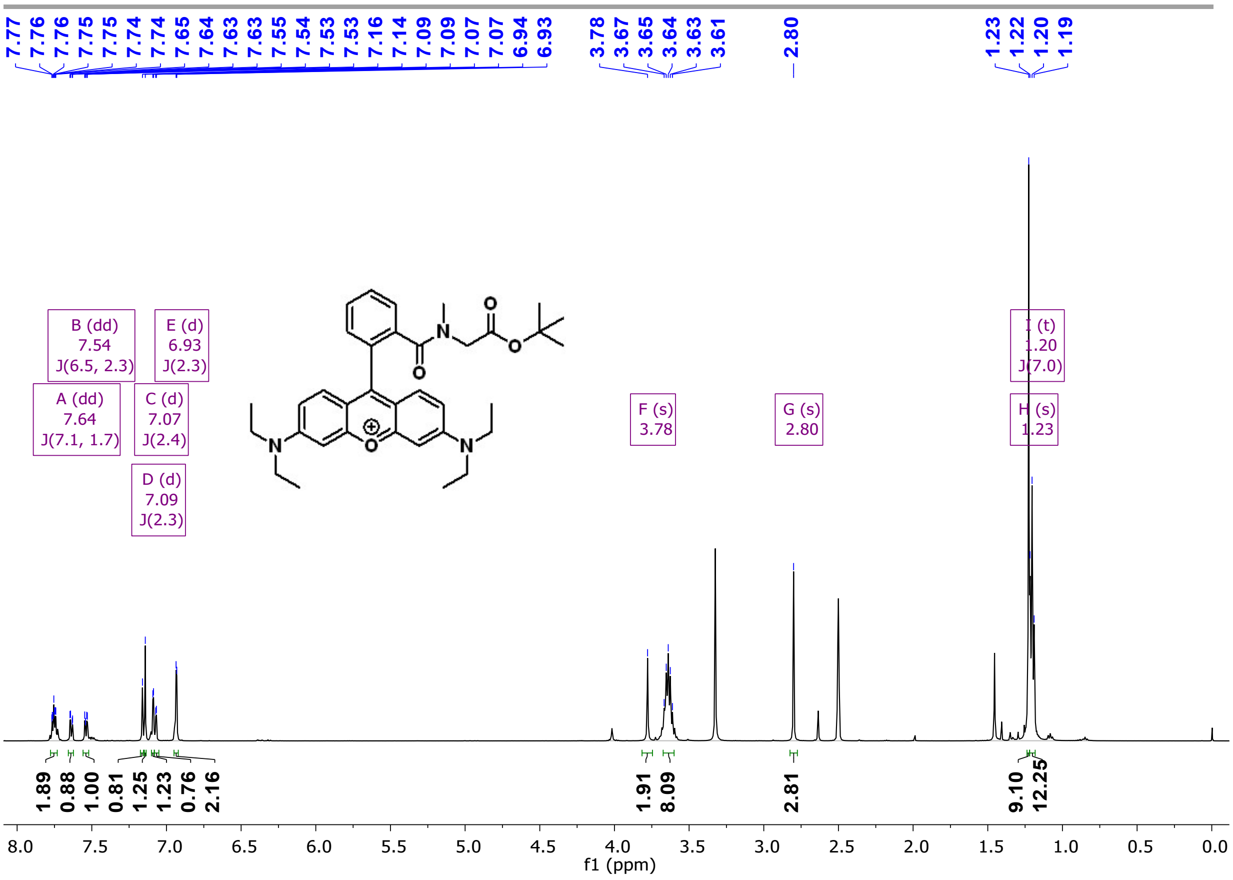

Figure S22. ^{1}H NMR spectrum of RhoB1 (500 MHz, in DMSO-d_6).

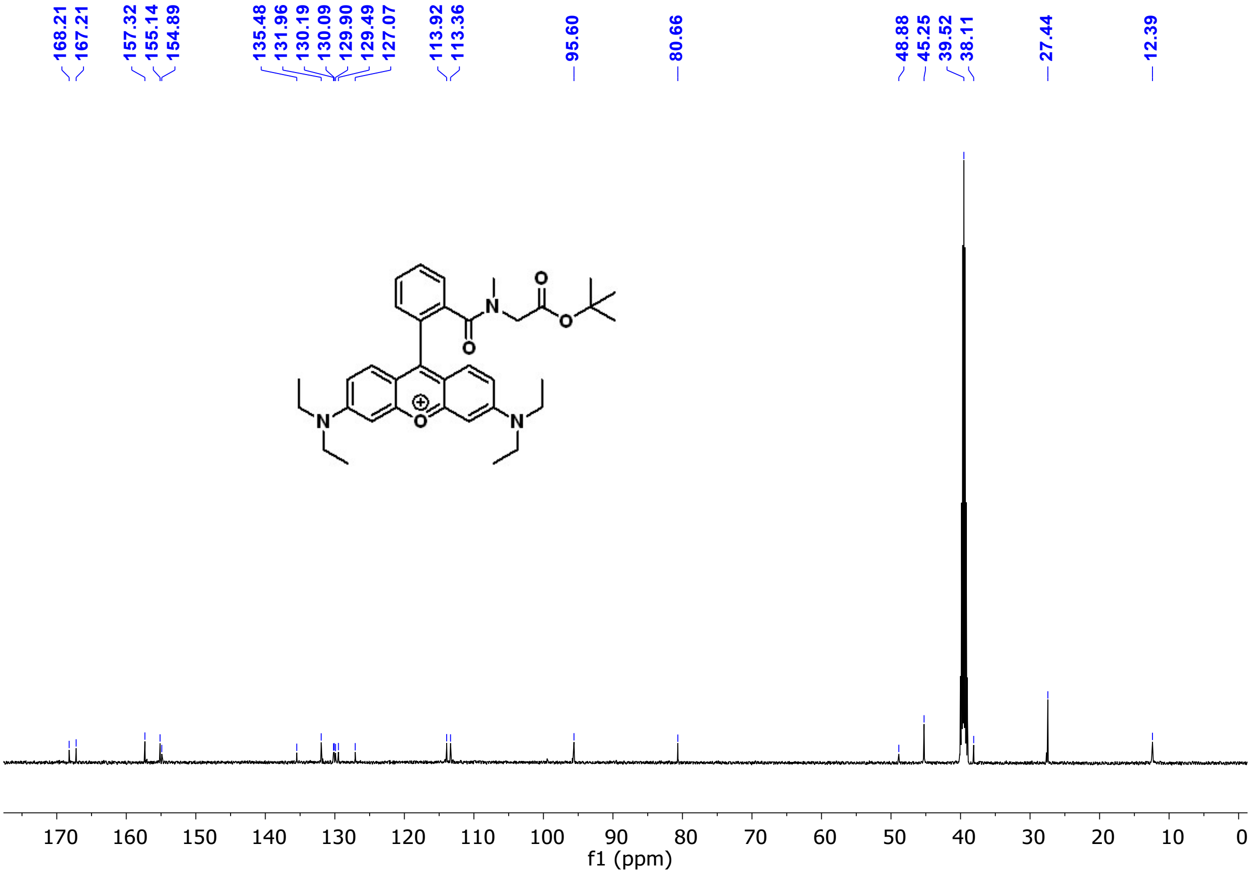

Figure S23. ^{13}C NMR spectrum of RhoB1 (126 MHz, in DMSO-d_6).

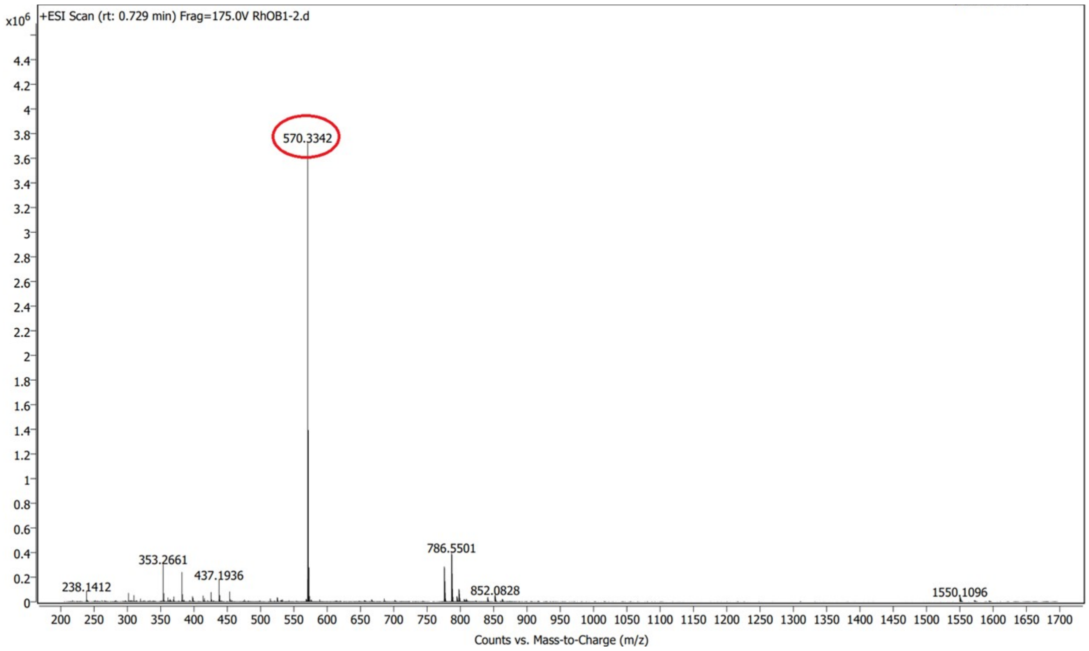

Figure S24. HRMS spectrum of RhoB1.

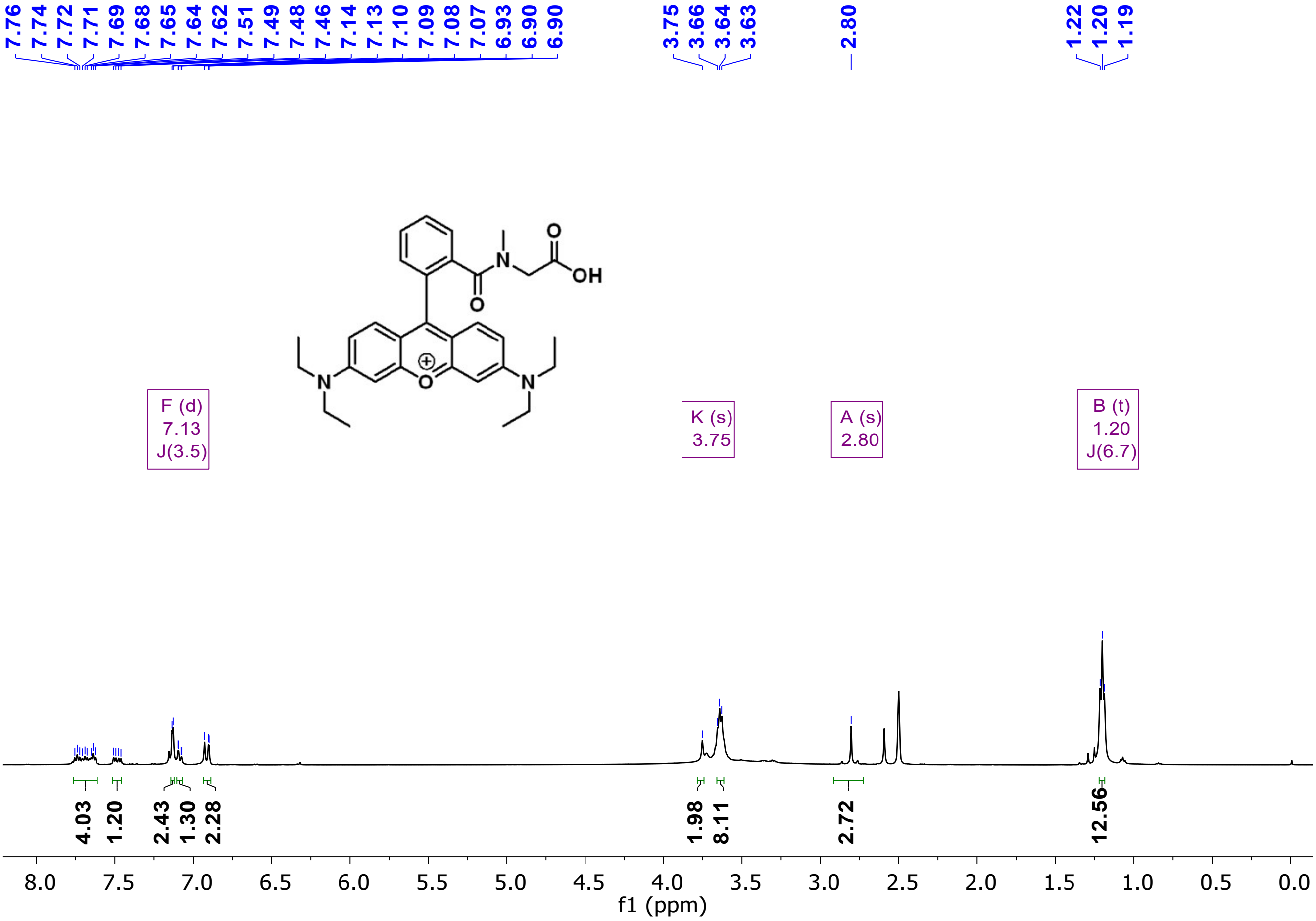

Figure S25. ^{1}H NMR spectrum of RhoB-Sar (500 MHz, in DMSO-d_6).

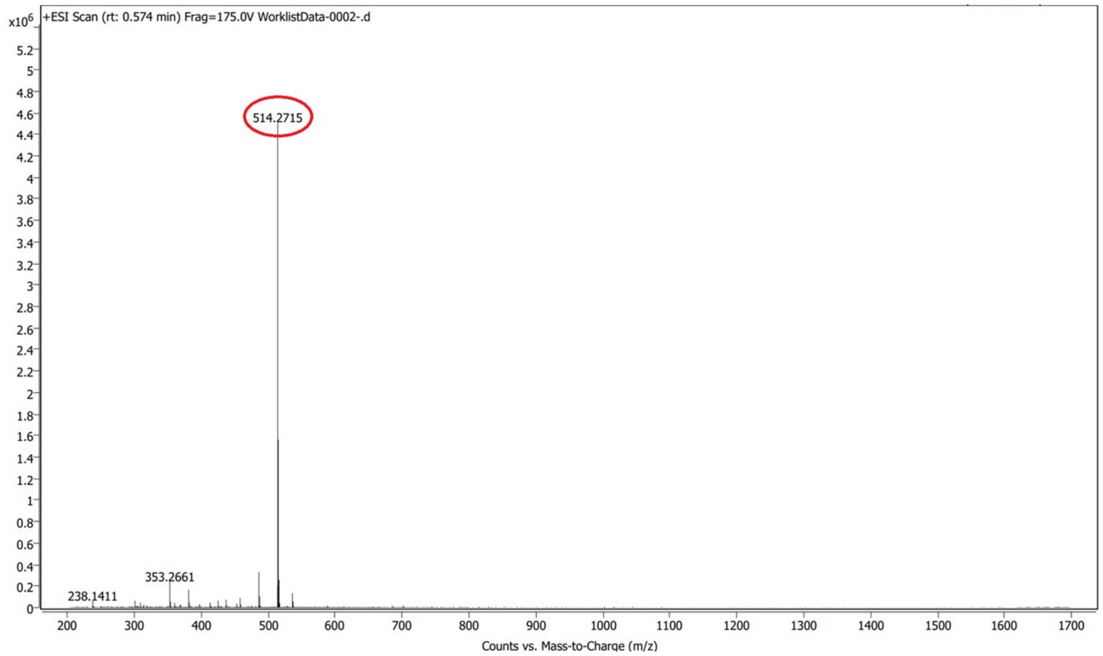

Figure S26. HRMS spectrum of RhoB-Sar.

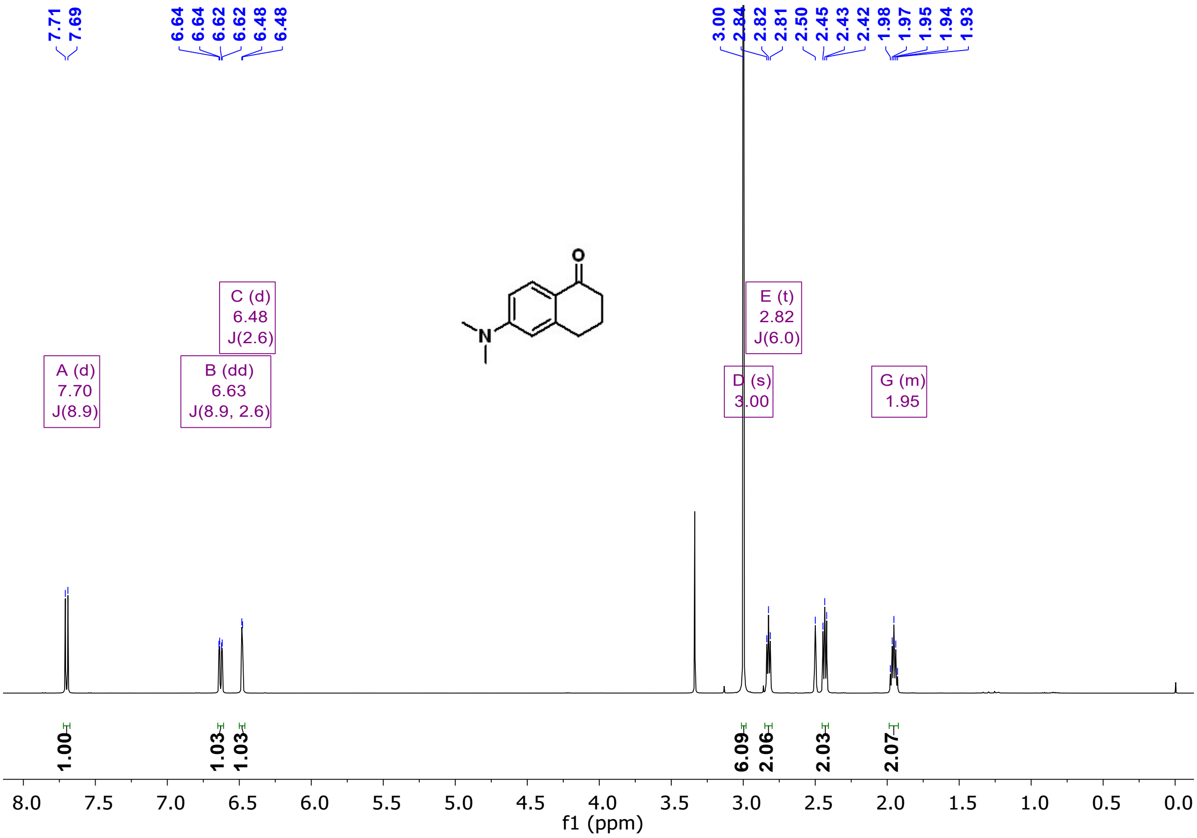

Figure S27. ^{1}H NMR spectrum of R1 (500 MHz, in DMSO-d_6).

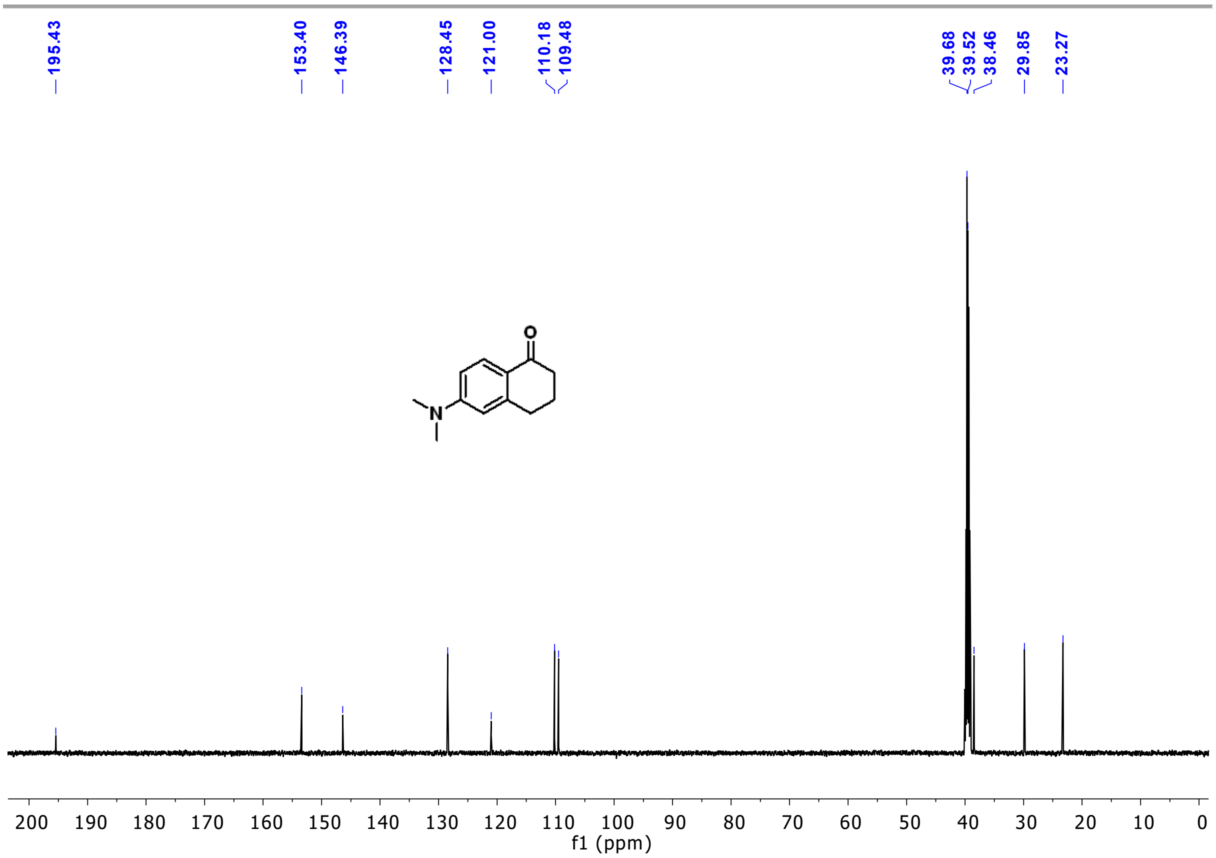

Figure S28. ^{13}C NMR spectrum of R1 (126 MHz, in DMSO-d_6).

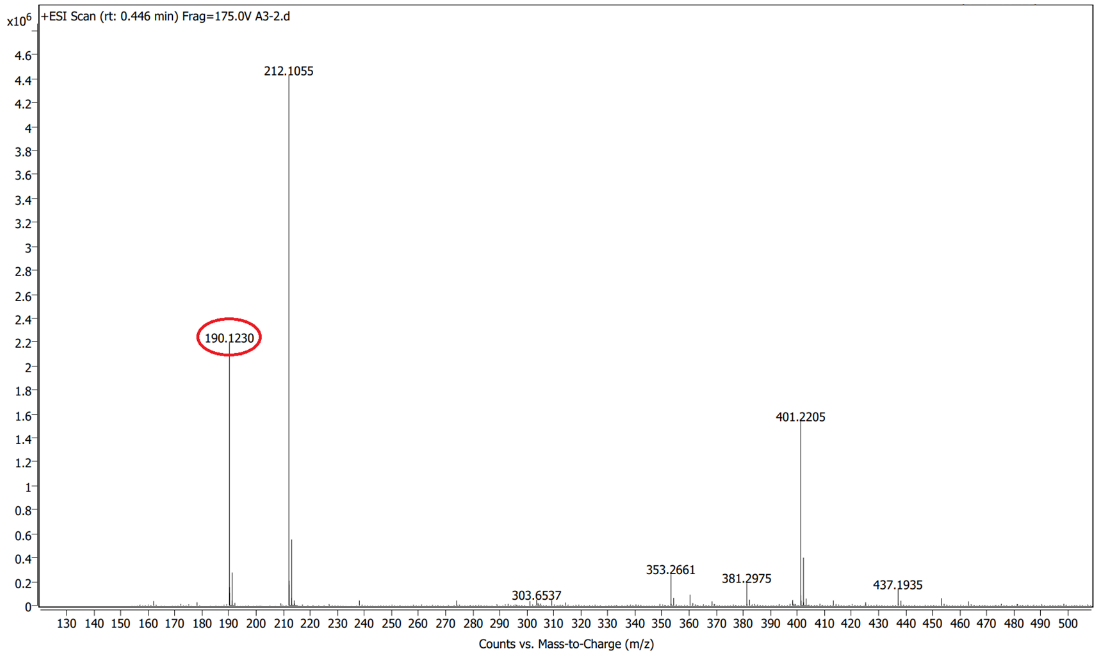

Figure S29. HRMS spectral analysis of R1.

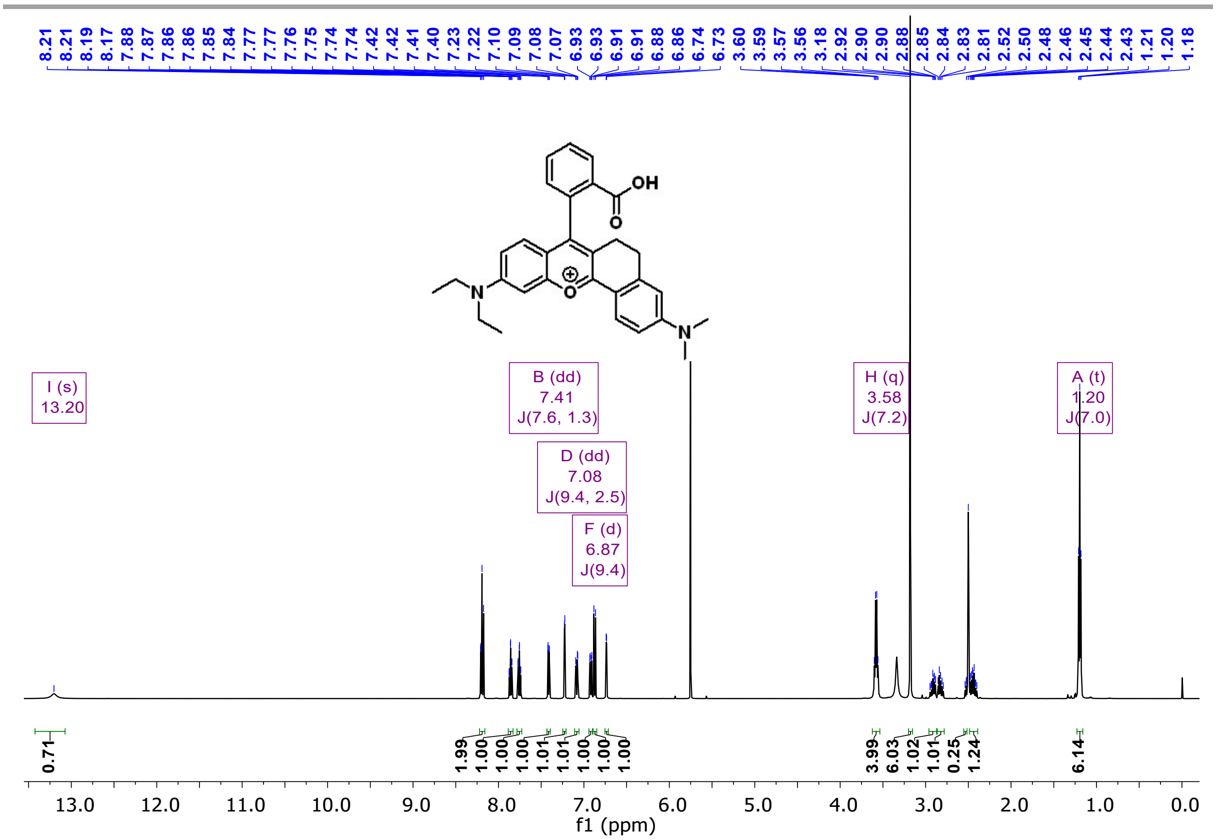

Figure S30. ^{1}H NMR spectrum of hNR (500 MHz, in DMSO-d_6).

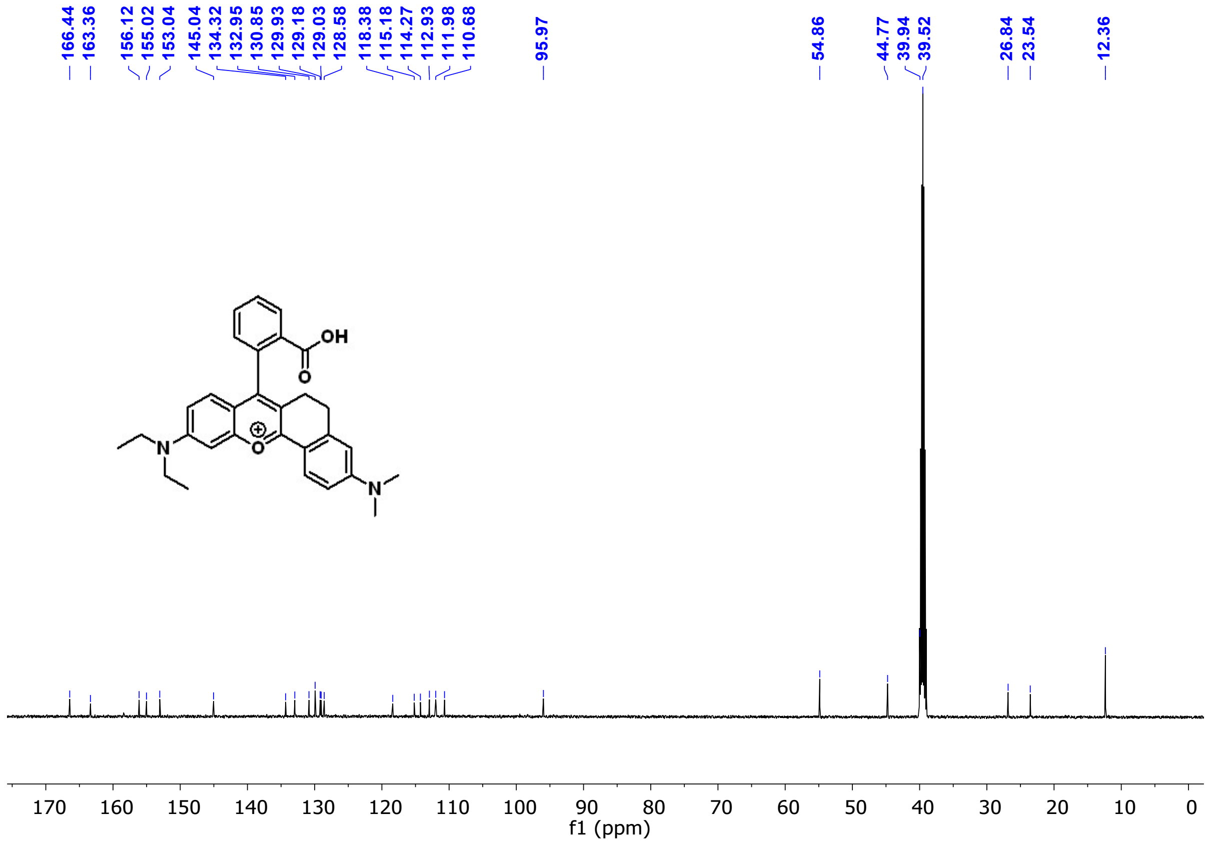

Figure S31. ^{13}C NMR spectrum of hNR (126 MHz, in DMSO-d_6).

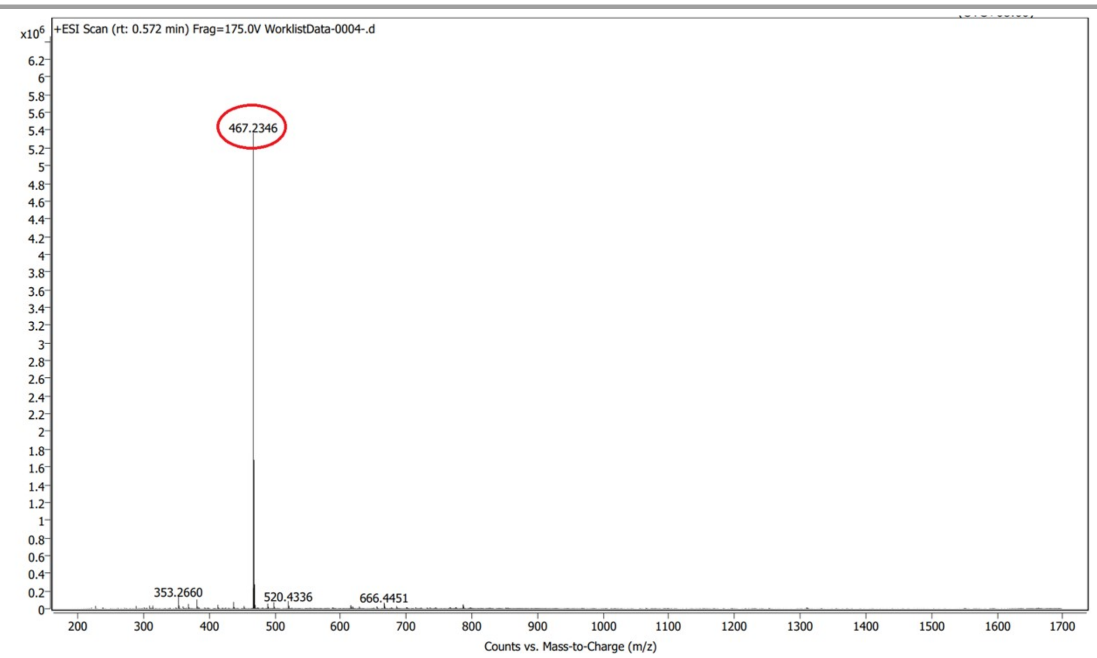

Figure S32. HRMS spectral analysis of hNR.

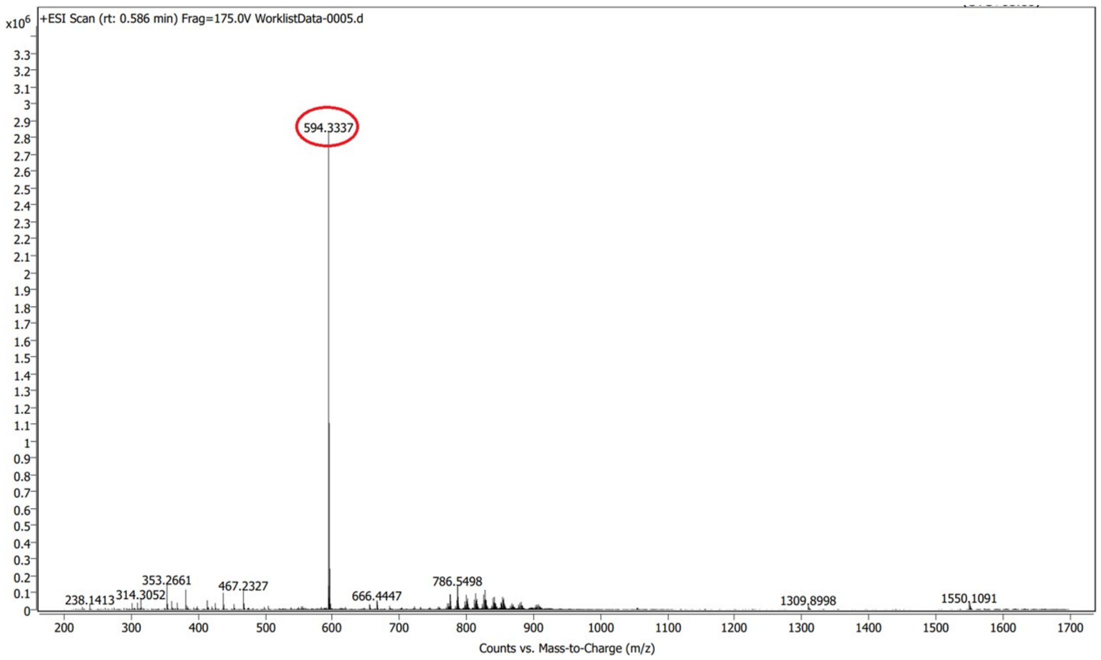

Figure S33. HRMS spectral analysis of hNR1.

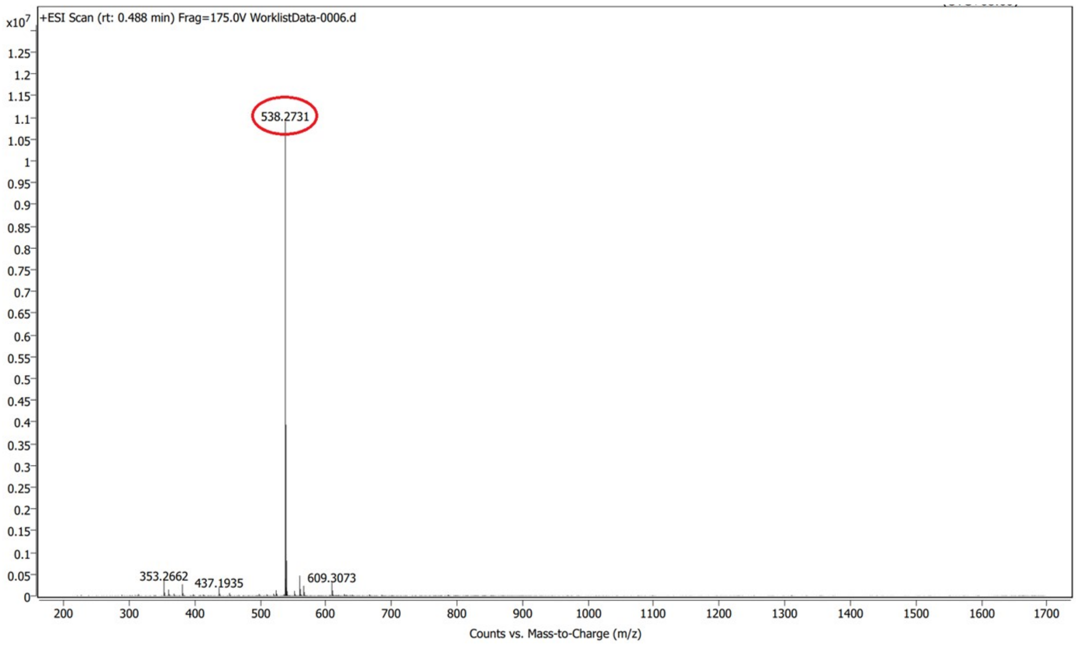

Figure S34. HRMS spectral analysis of hNR2.

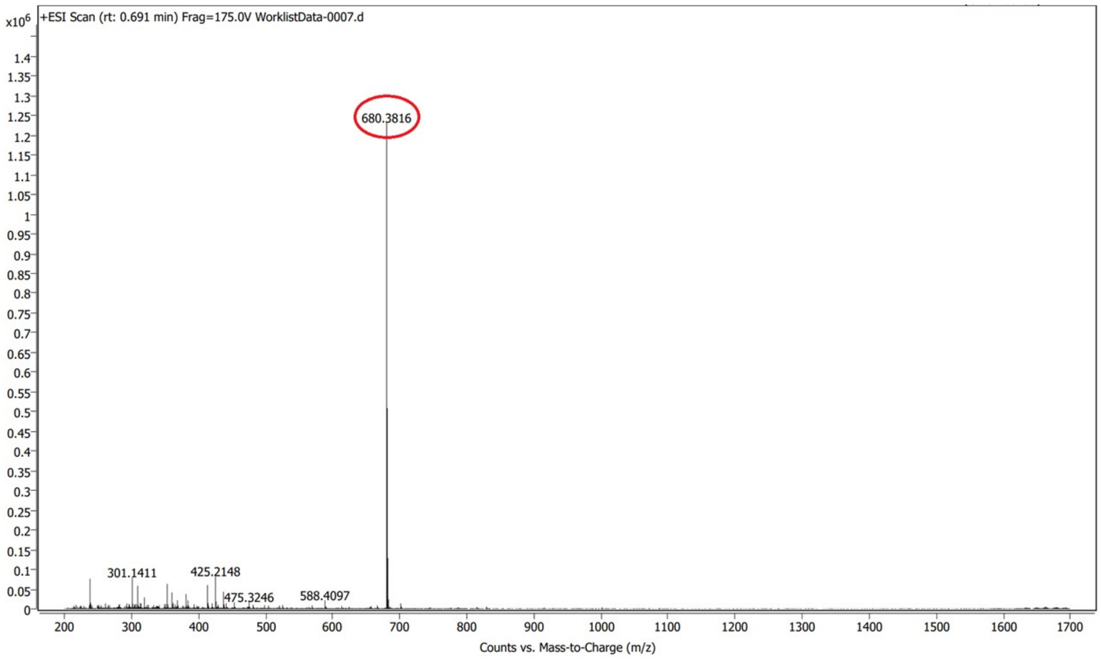

Figure S35. HRMS spectral analysis of hNR3.

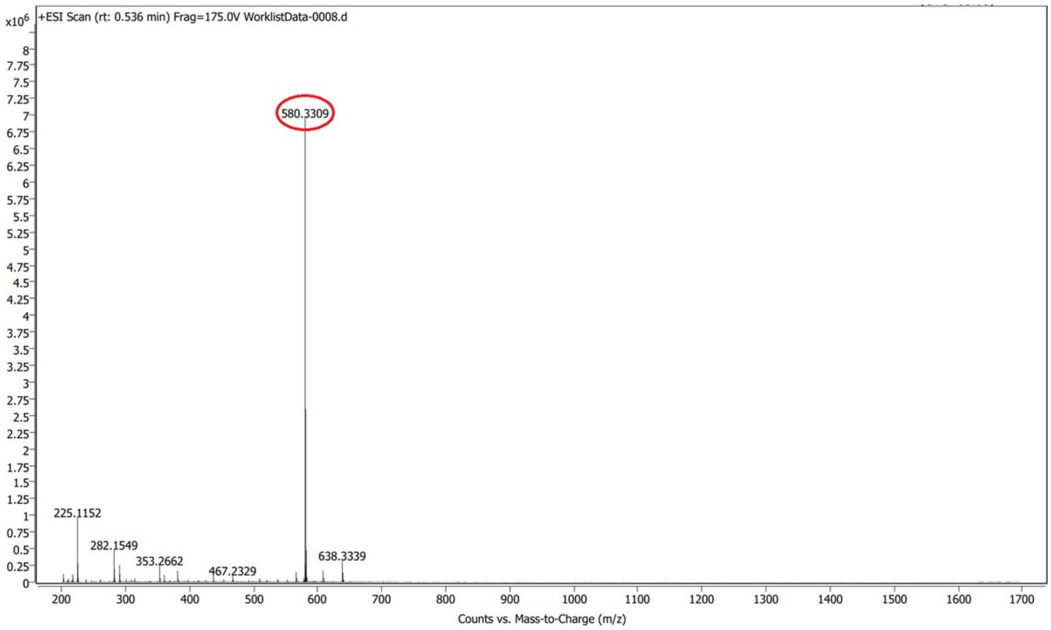

Figure S36. HRMS spectral analysis of hNR-NH_2.

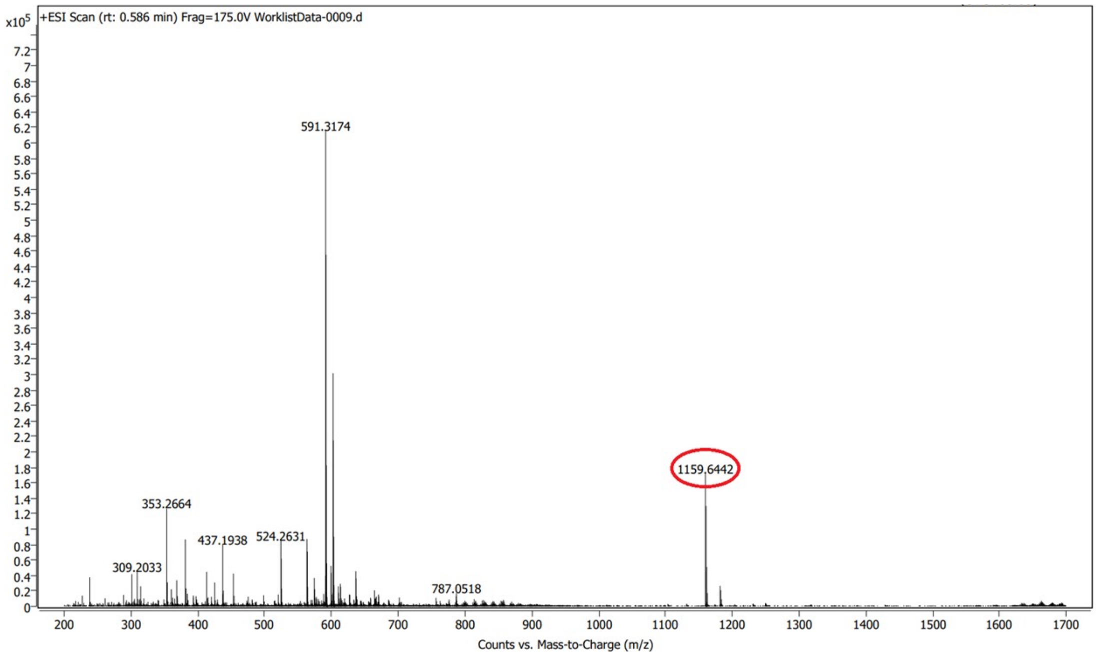

Figure S37. HRMS spectral analysis of Rho-FLTDG.

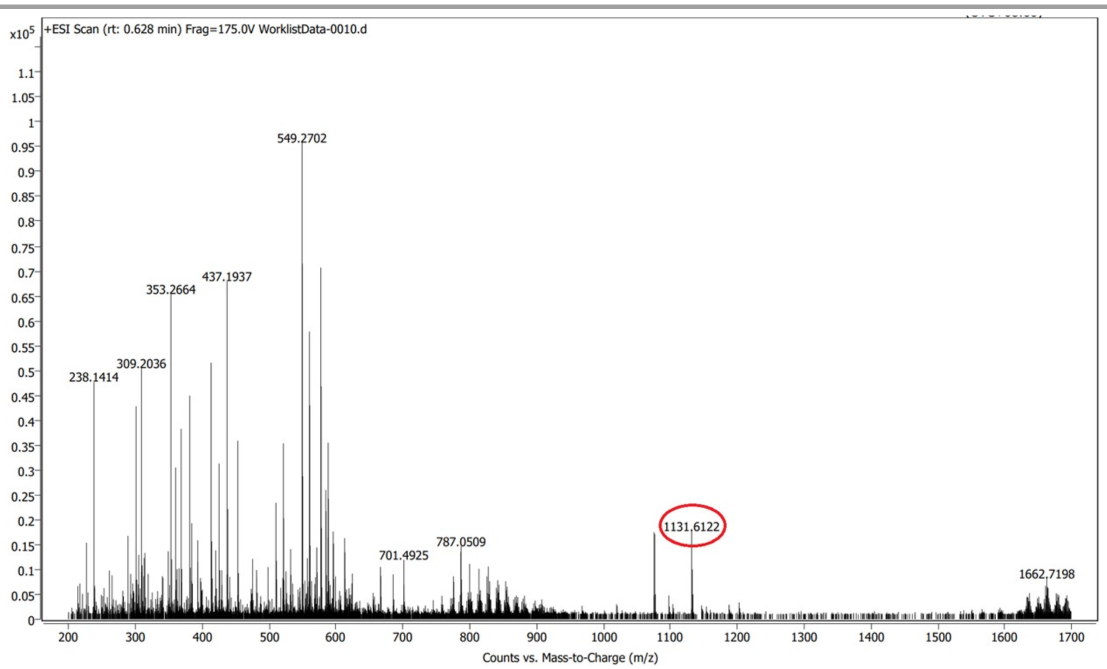

Figure S38. HRMS spectral analysis of Rho-YVADG.

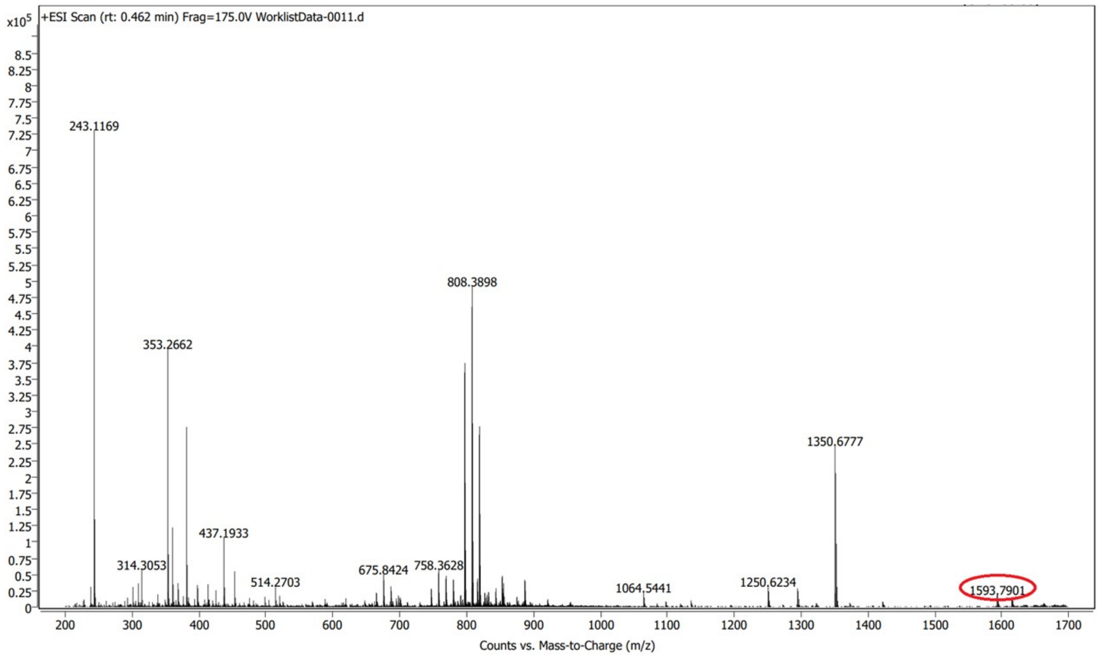

Figure S39. HRMS spectral analysis of Rho-WEHDG.

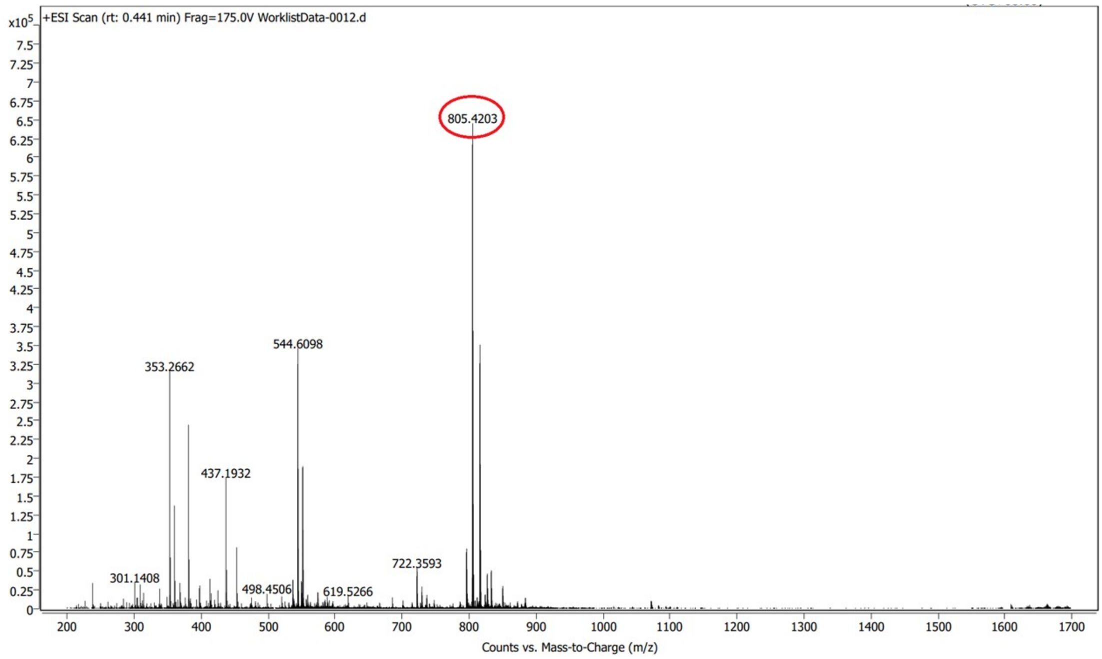

Figure S40. HRMS spectral analysis of **FPy1**.

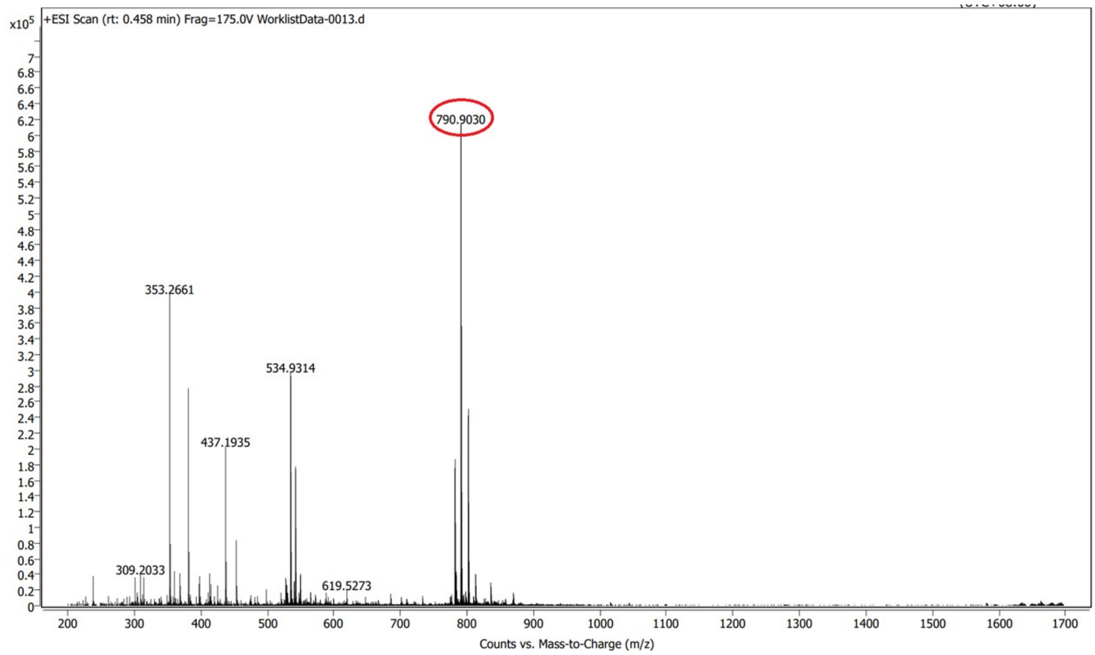

Figure S41. HRMS spectral analysis of **YPy1**.

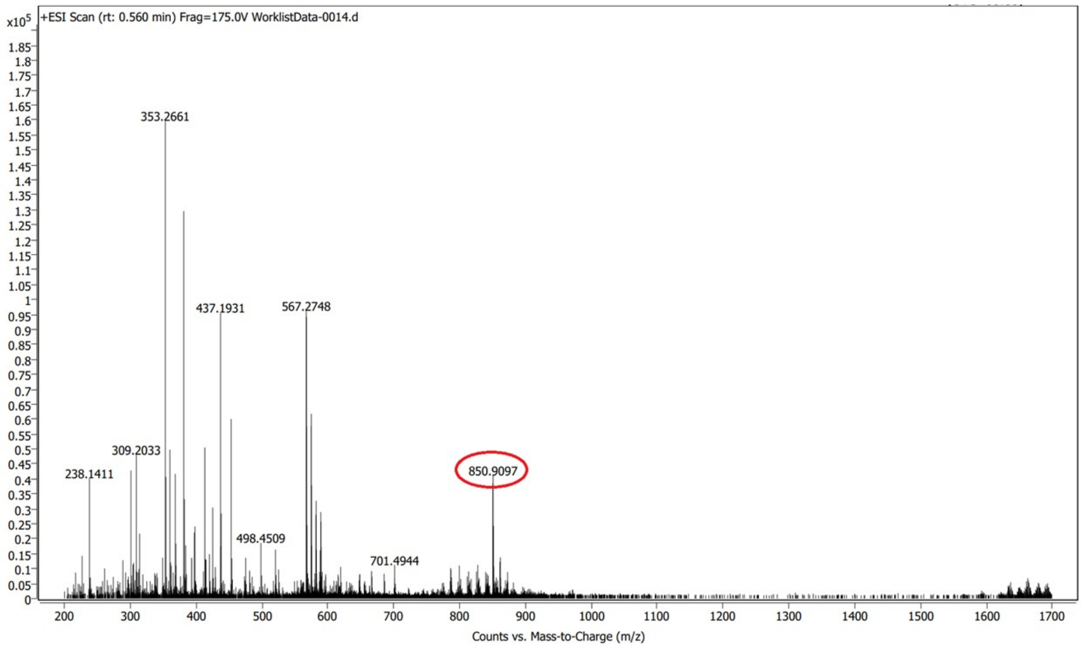

Figure S42. HRMS spectral analysis of **WPy1**.

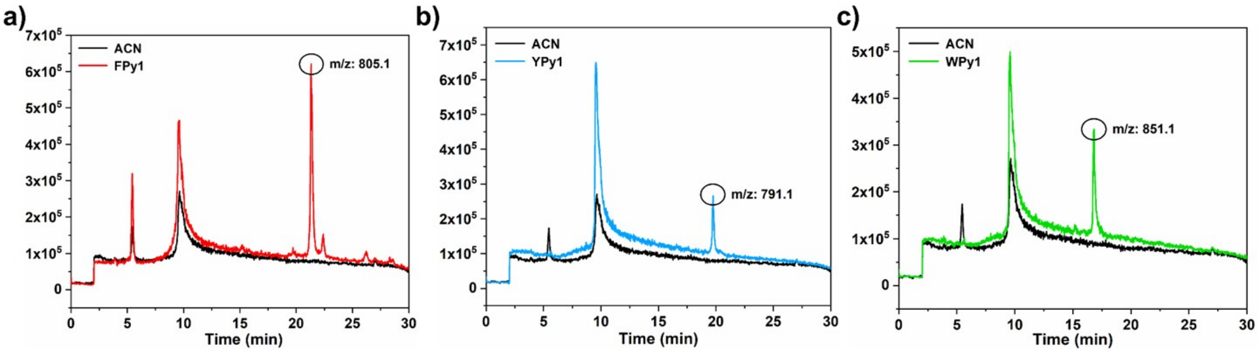

Figure S43. (a) LC-MSD analysis of **FPy1** (red) and blank acetonitrile (black); (b) LC-MSD analysis of **YPy1** (blue) and blank acetonitrile (black); (c) LC-MSD analysis of **WPy1** (green) and blank acetonitrile (black).
